# Palliative care for children: methodology for the development of a national clinical practice guideline

**DOI:** 10.1186/s12904-023-01293-3

**Published:** 2023-12-01

**Authors:** Kim C. van Teunenbroek, Leontien C. M. Kremer, A. A. Eduard Verhagen, Johannes M. A. Verheijden, Hester Rippen, Brigitt C. M. Borggreve, Erna M. C. Michiels, Renée L. Mulder, on behalf of the Dutch Paediatric Palliative Care Guideline Panel, Inge M. L. Ahout, Mattijs W. Alsem, Esther M. M. van den Bergh, Loes Berkhout, Karin G. C. B. Bindels-de Heus, Govert Brinkhorst, Arno Colenbrander, Linda Corel, Catharina M. Delsman-van Gelder, Jennifer van Dijk, Jurrianne C. Fahner, Jeannette L. Falkenburg, Laurent Favié, Annemie F. S. Galimont-Collen, Karin Geleijns, Rosa Geurtzen, Annelies Gijsbertsen-Kool, Saskia J. Gischler, Marinka A. R. de Groot, Anne Haag, Lisette ‘t Hart-Kerkhoffs, Hanneke Heinen, Katja M. J. Heitink-Polle, Petra Honig-Mazer, Carolien S. M. Huizinga, Cindy Joosen, Carla C. M. Juffermans, Marijke C. Kars, Karolien Kisman, Hennie Knoester, Eline M. Kochen, Barbara de Koning, Tom de Leeuw, Jeffry Looijestijn, Hilda Mekelenkamp, Maarten O. Mensink, Selma Mulder, Mariska P. Nieuweboer, Sebastianus B. J. Oude Ophuis, Suzanne G. M. A. Pasmans, Elise M. van de Putte, Emmy Räkers, Liesbeth Rietveld, Irma M. Rigter, Christel D. Rohrich, Tanja van Roosmalen, Elisabeth J. Ruijgrok, Jolanda H. Schieving, Kim van der Schoot, Antoinette Y. N. Schouten-van Meeteren, Ellen Siegers-Bennink, Henriette Sjouwke, Tanneke Snijders-Groenendijk, Mara van Stiphout, Suzanne van de Vathorst, Leo van Vlimmeren, Mirjam A. de Vos, Nellie van Wageningen, Anne Weenink, Willemien de Weerd, Ilse H. Zaal-Schuller

**Affiliations:** 1 Princess Máxima Centre for Pediatric Oncology, Utrecht, The Netherlands; 2 Department of Paediatrics, Emma Children’s Hospital, Amsterdam University Medical Centre (UMC), University of Amsterdam, Amsterdam, the Netherlands; 3 University Medical Centre Utrecht, Wilhelmina Children’s Hospital, Utrecht, the Netherlands; 4 Department of Paediatrics, Beatrix Children’s Hospital, University Medical Centre Groningen, University of Groningen, Groningen, the Netherlands; 5 Dutch Knowledge Centre for Children’s Palliative Care, Utrecht, the Netherlands; 6 Stichting Kind en Ziekenhuis, Utrecht, the Netherlands; 7 the Netherlands Comprehensive Cancer Organisation (IKNL), Utrecht, the Netherlands

**Keywords:** Clinical practice guideline, Evidence-based medicine, Paediatric palliative care

## Abstract

**Background:**

Provision of paediatric palliative care for children with life-threatening or life-limiting conditions and their families is often complex. Guidelines can support professionals to deliver high quality care. Stakeholders expressed the need to update the first Dutch paediatric palliative care guideline with new scientific literature and new topics. This paper provides an overview of the methodology that is used for the revision of the Dutch paediatric palliative care guideline and a brief presentation of the identified evidence.

**Methods:**

The revised paediatric palliative care guideline was developed with a multidisciplinary guideline panel of 72 experts in paediatric palliative care and nine (bereaved) parents of children with life-threatening or life-limiting conditions. The guideline covered multiple topics related to (refractory) symptom treatment, advance care planning and shared-decision making, organisation of care, psychosocial care, and loss and bereavement. We established six main working groups that formulated 38 clinical questions for which we identified evidence by updating two existing systematic literature searches. The GRADE (CERQual) methodology was used for appraisal of evidence. Furthermore, we searched for additional literature such as existing guidelines and textbooks to deal with lack of evidence.

**Results:**

The two systematic literature searches yielded a total of 29 RCTs or systematic reviews of RCTs on paediatric palliative care interventions and 22 qualitative studies on barriers and facilitators of advance care planning and shared decision-making. We identified evidence for 14 out of 38 clinical questions. Furthermore, we were able to select additional literature (29 guidelines, two textbooks, and 10 systematic reviews) to deal with lack of evidence.

**Conclusions:**

The revised Dutch paediatric palliative care guideline addresses many topics. However, there is limited evidence to base recommendations upon. Our methodology will combine the existing evidence in scientific literature, additional literature, expert knowledge, and perspectives of patients and their families to provide recommendations.

**Supplementary Information:**

The online version contains supplementary material available at 10.1186/s12904-023-01293-3.

## Background

In the Netherlands, each year 5000 to 7000 children aged 0 to 18 years are suffering from life-threatening or life-limiting conditions [1]. In 2021, 983 children, adolescents and young adults aged 0 to 20 years died due to the consequences of these conditions [2]. Although these numbers seem small, the impact of these diseases for children and their families involved, is immeasurable. All these children and their families need paediatric palliative care.

Paediatric palliative care is a specialty that encompasses the care of all children with life-threatening or life-limiting conditions regardless of their diagnosis or stage of disease [3]. The World Health Organization (WHO) defines paediatric palliative care as the prevention and relief of suffering of paediatric patients and their families, facing problems associated with life-threatening or life-limiting conditions [4]. These problems include the physical, psychological, social and spiritual suffering of children and the psychological, social and spiritual suffering of family members [4]. Thus, paediatric palliative care relates not only to the child but to the whole family [5].

Both health care providers and parents face multiple challenges in providing the best paediatric palliative care, as it is a complex trajectory where curative and palliative care intertwine. Additionally, the child continues to develop physically, emotionally and cognitively which contributes to many varieties in the child’s communication skills and ability to understand the condition [5]. Since the number of children living with life-threatening or life-limiting conditions is small and many conditions are extremely rare or specific to childhood, there is only limited knowledge on paediatric palliative care [5].

To ensure that all children in need of palliative care receive high quality care, clinical practice guidelines (CPGs) are needed. Care consistent with CPGs has led to more efficient care delivery and improved patient outcomes [6–8]. Therefore, the Dutch multidisciplinary CPG for paediatric palliative care was developed in 2013. This CPG provides recommendations on symptom relief, decision-making and organisation of care. Several years after the development of the first national CPG, stakeholders expressed a need to update this CPG with newly published evidence and to include new topics, specifically delirium, palliative sedation, restriction of hydration and nutrition in case of palliative sedation, advance care planning, shared decision-making, psychosocial care, and loss and bereavement. As a result, the revision of the first Dutch CPG for paediatric palliative care was initiated.

This article provides an overview of the methodology for this revision of the Dutch CPG for paediatric palliative care and provides a brief presentation of the identified evidence. In subsequent manuscripts we will discuss the evidence and recommendations on (1) treatment of symptoms including refractory symptoms, (2) advance care planning, shared decision-making, and organisation of care and (3) psychosocial care, and loss and bereavement.

## Methods

### Aim and scope

The aim of this CPG is to provide guidance on all aspects of palliative care including physical, psychological, social, and spiritual aspects, for all children aged 0 to 18 years with life-threatening or life-limiting conditions and their caregivers, brothers, and sisters (hereafter referred to as families) throughout the entire palliative trajectory (from palliative diagnosis till after end-of-life), with the ultimate goal to improve quality of paediatric palliative care. This CPG is intended for all health care providers from different specialisms who are involved in paediatric palliative care and for all children aged 0 to 18 years with life-threatening or life-limiting conditions and their families.

The guideline is an update of the first CPG for paediatric palliative care that was published in 2013, which provided recommendations on symptom relief, decision making and organisation of care.

### Topic selection

In 2018, an invitational conference among experts in paediatric palliative care was convened to evaluate the first CPG for paediatric palliative care and identify new topics that needed to be addressed. This formed the basis for the online survey that was conducted among 89 health care providers to prioritize new topics (Appendix A). In this survey, professionals were asked to weigh topics that should be included during the revision of the CPG on a 5-point Likert scale that ranged from not important to very important. In addition, another survey was conducted among patient representatives and mostly bereaved parents of children with life-threatening or life-limiting conditions (n = 16) to indicate their priorities towards the identified topics in the invitational conference (Appendix B).

**Fig. 1:**
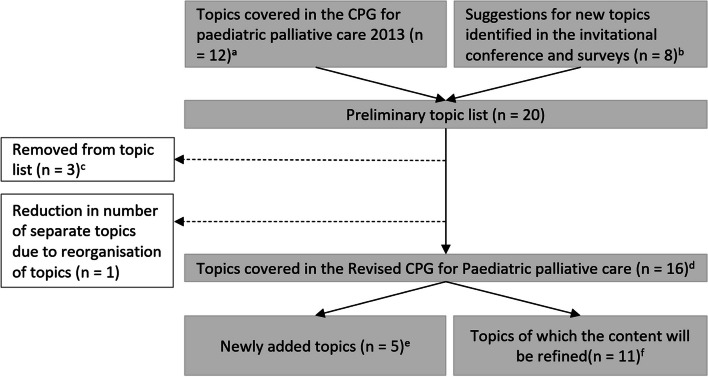
Process of topic selection **a** Topics covered in the CPG for paediatric palliative care 2013 are: anxiety and depression; dyspnoea; haematological symptoms; coughing; skin complaints; nausea and vomiting; neurological symptoms; pain; death rattle; fatigue; decision making; and organisation of care.  **b** Suggestions for topics identified in the invitational conference and surveys are: delirium; refractory symptoms; psychosocial care; loss and bereavement; advance care planning; practicing communication skills; financing; and complementary care. **c** Reasons for removal from topic list are: practicing communication skills and financing are outside the scope of this CPG; and, complementary care will be covered in another CPG. **d** Topics covered in the revised CPG for paediatric palliative care are: anxiety and depression; delirium; dyspnoea; haematological symptoms; coughing; skin complaints; nausea and vomiting; neurological symptoms; pain; death rattle; fatigue; refractory symptoms; advance care planning and shared decision making; organisation of care; psychosocial care; and loss and bereavement.  **e** Newly added topics are: delirium; refractory symptoms; advance care planning and shared decision making; psychosocial care; and loss and bereavement.  **f** Topics of which the content will be refined: anxiety and depression; dyspnoea; haematological symptoms; coughing; skin complaints; nausea and vomiting; neurological symptoms; pain; death rattle; fatigue; and organisation of care

Multiple suggestions for new topics were derived from the results of the invitational conference and surveys. A preliminary topic list was generated which included topics covered in the CPG of 2013 (n = 12) and the suggestions for new topics (n = 8). The core group made a final selection based on practical and financial feasibility, and priorities of professionals, and patient and parent representatives. This resulted in a final list of 16 topics of which five topics were newly added. Figure 1 shows the selection process including reasons for exclusion of topics.

### Multidisciplinary guideline development panel

A guideline development panel was established which consisted of 72 experts in paediatric palliative care and nine (bereaved) parents (see representation of patients and their families). Professionals from various disciplines such as paediatricians, paediatric oncologists, neurologists, anaesthesiologists, nurses, psychologists, pharmacists, medical pedagogical care providers and researchers, were included in the expert panel. Each working group (WG) consisted of members with expertise knowledge relevant to the specific topic addressed (Appendix C). The WG members were selected based on their experience with paediatric palliative care, of whom some had specific certified training in paediatric palliative care. The Netherlands Comprehensive Cancer Organisation recruited the panel members. Members were either mandated by their professional associations or participated on personal title. All members disclosed conflicts of interest at the start and end of the guideline development process.

**Fig. 2:**
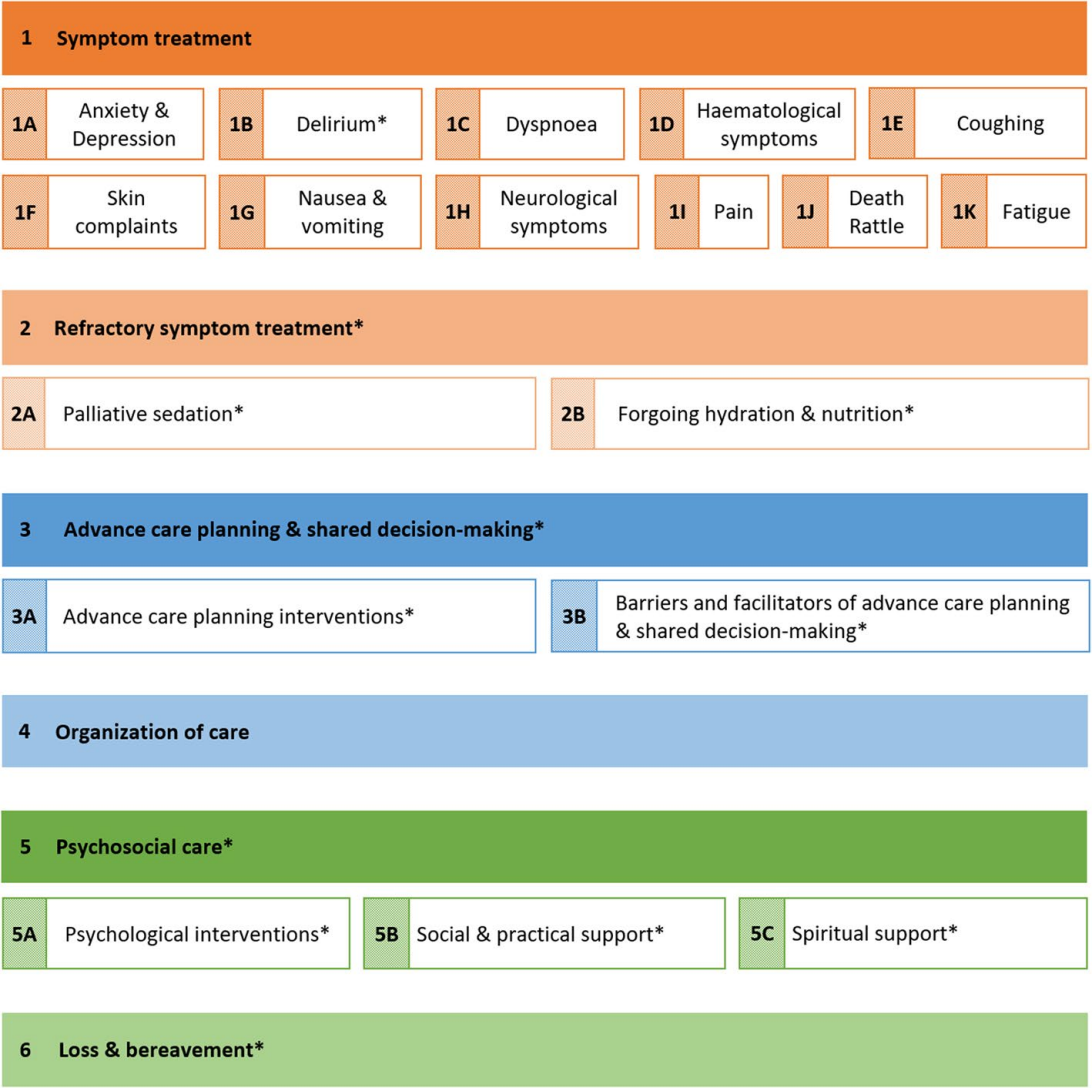
Guideline working groups * Newly added in the revision of the Dutch Paediatric Palliative Care CPG

Based on the final selection of topics, six main WGs were formed. These WGs focused on symptom treatment (WG1), refractory symptom treatment (WG2), advance care planning and shared decision-making (WG3), organisation of care (WG4), psychosocial care (WG5), and loss and bereavement (WG6). Sub-WGs were established for WGs that covered multiple topics. Figure 2 provides a full overview of the (sub)WGs. The members of the expert panel were appointed to the (sub)WGs according to their expertise. Moreover, a core group was established to ensure consistency and transparency throughout the guideline. An overview of the working structure and guideline development process is shown in Appendix D and E.

### Representation of patients and their families

Different methods were used to ensure representation of patients and their families. First, we conducted a survey to identify patient priorities for topic selection. Second, two members of the core group were dedicated to ensure the representation of patients and their families during the entire guideline process. Third, a panel consisting of nine (bereaved) parents of children with life-threatening or life-limiting conditions was established to review guideline texts and recommendations (Appendix C). We ensured the panel represented a broad spectrum of experiences regarding paediatric palliative care by including parents of children with a variety of palliative conditions, age, and stage of disease (currently receiving palliative care or deceased). The parents were recruited by the Child and Hospital Foundation and attended a short training on guideline development.

The panel reviewed the first drafts of all guideline texts and recommendations. Additionally, the panel reviewed the complete concept guideline to ensure their input was incorporated correctly. Lastly, parents were asked to share their experiences during the interactive conference for organisation of care (WG4) (see consensus-based approach).

### Formulation of clinical questions

Each (sub-)WG proposed several clinically relevant questions. Questions were developed according to the PICOS format, which defines the patient group, intervention, comparison to the intervention, relevant outcomes, and study design for each clinical question. The core group assessed all clinical questions carefully. If necessary, clinical questions were adjusted. The core group sent the final clinical questions to the (sub-)WGs for approval.

Below we describe the methods used to answer the clinical questions. In Table 1, an overview of the methods used per WG is presented.

**Table 1:**
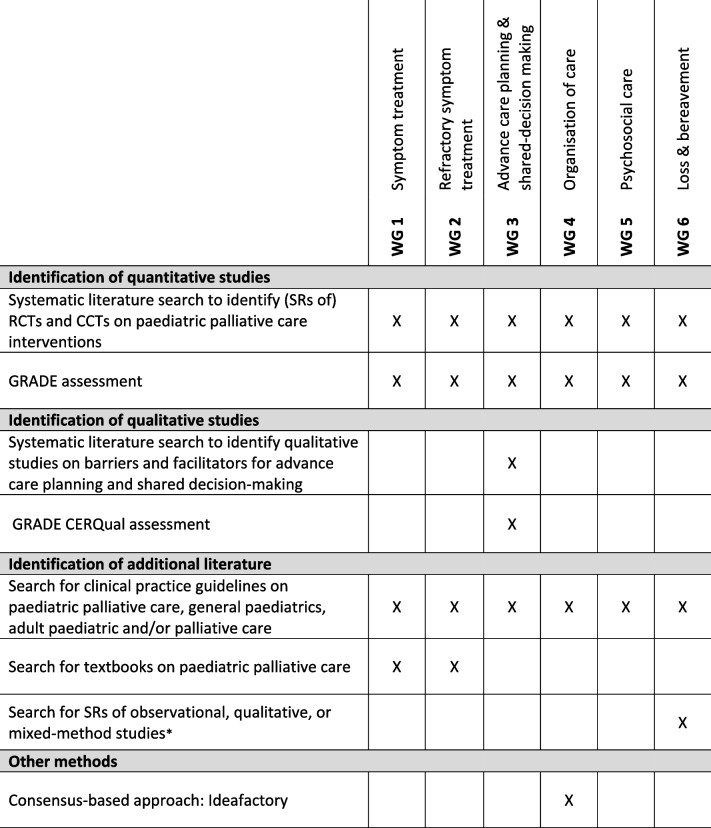
Overview of methods used per working group

^a^Studies are derived from the search for (SRs of) RCTS/CCTs on paediatric palliative care interventions

### Identification of evidence – quantitative studies

#### Systematic literature search

WG1 to WG6 formulated a total 37 clinical questions related to the effect of paediatric palliative care interventions (Appendix F).

We updated the systematic literature search of the former CPG (which searched from 1970 to 2011) [9] to identify new studies on paediatric palliative care. All originally included studies were also included in the revised CPG. We searched for studies published from January 1, 2010 to January 24, 2020 (initial search October 5, 2018; top-up search, January 24, 2020), in the databases Ovid MEDLINE and PreMEDLINE, MEDLINE (PubMed), CENTRAL and the Cochrane Database of systematic reviews using a combination of the search terms “child”, “palliative care”, “randomized controlled trial” and “systematic review” (Appendix G).

The following inclusion criteria were defined: (1) randomized controlled trials (RCTs), controlled clinical trials (CCTs) including at least 10 patients and systematic reviews (SRs) of RCTs, (2) study population consisting of children aged 0 to 18 with life-threatening conditions and life-limiting conditions (according to the definition of the WHO [4]); at least 75% of the study population should be aged 0 to 18 years, (3) paediatric palliative care interventions related to (a) treatment of anxiety and depression, delirium, dyspnoea, haematological symptoms, coughing, skin complaints, nausea and vomiting, pain, neurological symptoms and fatigue, (b) treatment of refractory symptoms, (c) advance care planning and shared decision-making, (d) organisation of care, (e) psychosocial care, and (f) loss and bereavement. Only studies published in English or Dutch language were included. Studies that described interventions on complementary or alternative medicine were excluded (Appendix H).

We searched for eligible studies in reference lists of included studies and identified SRs, guidelines, and textbooks. Moreover, we asked WG members to provide eligible studies.

#### Study selection

The studies were selected through two rounds of screening, title/abstract screening, and full text screening. One independent reviewer screened the titles and/or abstracts of all citations. The full text screening was performed by one independent reviewer. In case of doubt, the citations were discussed in the core group and were included only if there was consensus. The selected citations were distributed among the WGs. When citations were relevant for multiple WGs, they were included in all relevant WGs.

#### Summary and appraisal of evidence

All studies were summarized in evidence tables. Evidence tables described study characteristics (study type, setting, duration and years), participant characteristics (number and diagnosis of participants, age, and sex), intervention and control characteristics, outcomes and results, and strengths, limitations, and study quality.

We determined individual study quality by assessing risk of bias according to the criteria of Cochrane Risk of Bias tool [10]. This tool assesses risk of selection bias, attrition bias, detection bias and performance bias of each study. Risk of bias can be classified as either low, high, or unclear.

We categorized evidence by outcome measures in summary of findings tables for every clinical question. We then formulated conclusions of evidence for each outcome measure. We assessed the quality of the total body of evidence with the Grading Recommendation Assessment Development and Evaluation (GRADE) criteria [11]. The GRADE appraisal was performed by two reviewers. Quality of evidence was downgraded if study limitations, inconsistency, indirectness, imprecision, or publication bias were present. Quality of evidence was upgraded if a dose response effect or large magnitude of effect was identified.

### Identification of evidence – qualitative studies

#### Systematic literature search

WG3 (advance care planning and shared decision-making) formulated one clinical question on barriers and facilitators of advance care planning and shared decision-making (Appendix F). We performed a systematic literature search to identify qualitative studies on barriers and facilitators for advance care planning and shared decision-making. We updated the literature search that was conducted in the guideline ‘End of life care for infants, children and young people with life-limiting conditions (2016)’ of the National Institute for Health and Care Excellence (NICE) [12]. We searched Medline (PubMed) from January 1, 2016 to September 16, 2020 using the search terms “child”, “palliative care”, “advance care planning”, “shared decision-making”, “qualitative study” (Appendix G).

The following inclusion criteria were defined: (1) qualitative studies, mixed-methods observational studies with qualitative data and SRs of qualitative studies, (2) study population consisting of children aged 0 to 18 years old with life-threatening or life-limiting conditions, their parents and health care providers, (3) study outcomes were barriers and facilitators on advance care planning or shared decision-making. Moreover, only studies published in English or Dutch language were included (Appendix H).

We asked WG members to provide eligible studies and searched for eligible studies in identified SRs and guidelines on barriers facilitator for advance care planning and shared decision-making.

#### Study selection

Both title/abstract screening and full text screening were performed by two independent reviewers. One reviewer performed title/abstract screening and full text screening for all identified citations. For the second review, citations were divided among eight WG members. In case of doubt, citations were discussed in the core group and included only if there was consensus.

#### Summary and appraisal of evidence

All studies were summarized in evidence tables. Evidence tables described study characteristics (study type, objective, setting, duration, and years), participant characteristics (number and diagnosis of participants, age, sex, ethnicity, religious preference, and level of education), outcomes and results, and strengths, limitations, and study quality.

We determined individual study quality by assessing the methodological limitations according to the criteria of Critical Appraisal Skills Programme (CASP) checklist tool [13]. This tool assesses the aim and appropriateness of the qualitative study design, rigor in study design, sample selection, data collection, data analysis and results. Methodological limitations are classified as low, high, or unclear.

We assessed the quality the total body of evidence with the adapted GRADE Confidence in the Evidence from Reviews of Qualitative research (GRADE CERQual) methodology [14]. The GRADE CERQual appraisal was performed by two reviewers. Quality of evidence was downgraded if methodological limitations were present or if there was a lack of coherence, relevance or data saturation [15]. Quality of each conclusion of evidence was classified as high, moderate, low, or very low.

### Identification of additional literature

As the expectation was that the systematic searches would yield little to no evidence, we searched for additional literature.

For all WGs, we searched for guidelines on paediatric palliative care, general paediatrics, and adult palliative care. To identify relevant (inter)national guidelines on paediatric palliative care, general paediatrics, and adult palliative care, we searched multiple databases. We searched the Guideline International Network database from 2010 to January 24, 2020, using the search terms “child” and “palliative care”, to identify guidelines on paediatric palliative care. Furthermore, we searched databases of the NICE, International Paediatric Oncology Guidelines in supportive care network (iPOG), the Dutch Association for Paediatrics, and Pallialine to identify guidelines on paediatric palliative care, general paediatrics, and adult palliative care. Guideline panel members were also asked to supply additional guidelines (Appendix G).

For the selection of guidelines, our first choice was to include(inter)national guidelines on paediatric palliative care. If guidelines on paediatric palliative care were not available, we included guidelines on general paediatrics if deemed relevant such as for topics related to (refractory) symptom treatment. We only included guidelines on adult palliative care, if no (relevant) guidelines on paediatric palliative care or guidelines on general paediatrics were identified (Appendix H). Throughout the guideline process, we manually checked for all selected guidelines if more recent versions were available.

In case we did not find recommendations from guidelines on paediatric palliative care, general paediatrics, or adult palliative care, we included two textbooks on paediatric palliative care to refine considerations and recommendations. Since most topics were covered in recommendations from selected guidelines, we only used the textbooks for WG1 (symptom treatment) and WG2 (refractory symptom treatment).

Lastly, we derived SRs of observational, qualitative, or mixed-method studies from the systematic literature search on paediatric palliative care and through referencing. The inclusion of these SRs was only considered relevant for WG6 (loss and bereavement). We summarized the results of the SRs in evidence tables and translated these into conclusions of evidence. As the results of SRs included multiple studies from multiple study designs, we were not able to determine individual study quality nor the quality of the total body of evidence. The formulated conclusions were used to base recommendations upon.

### Consensus-based approach

We found that not all included clinical questions in the revised CPG could be appropriately answered through an evidence-based approach, as some questions were considered as highly specific to the Dutch context. In particular, WG4 (organisation of care) formulated clinical questions that focused on issues specific to the Dutch health care system and professional roles and institutions within this system (for example, methods to assist the general practitioner and other health care providers in improving continuity of paediatric palliative care at home). Therefore, an Ideafactory was organized. This is an interactive conference with a competitive element that is designed to find the best solutions for problems (formulated as questions). These solutions were used as the basis for formulating the recommendations. The methods and results will be presented in a subsequent manuscript.

### Formulation of recommendations

When formulating recommendations, several factors were taken into account: (1) the quality of the evidence (the higher the quality, the more likely it is to formulate a strong recommendation), (2) additional literature, (3) patient perspectives (values and needs), (4) professional perspectives (clinical expertise, values and needs), (5) acceptability (legal and ethical considerations), (6) feasibility (sufficient time, knowledge and manpower) and (7) benefits versus harms of the interventions.

For each clinical question, WG members described the relevant considerations. Decisions were made through group consensus. The strength of each recommendation was graded according to published evidence-based methods [16, 17] (Appendix I). Recommendations were categorised as strong to do (green), moderate to do (yellow) or strong not to do (red). A strong recommendation reflected a high degree of certainty. Moderate recommendations have a higher degree of uncertainty, therefore factors such as the clinical expertise, the patients and family’s situation and preferences, feasibility and relevant harms and benefits need to be considered.

All recommendations were supported unanimously by the core group, WG members, and parent representatives.

## Results

### Identification of quantitative studies

For the 37 formulated clinical questions on paediatric palliative care interventions of WG1 to WG6, the updated systematic literature search yielded 5078 citations. A total of 4337 citations were excluded based on title/abstracts and 168 citations were included for full text screening. Main reasons for exclusion of full texts were wrong study design (other than RCTs, CCTs or SRs) or wrong study population (other than children in the palliative setting).

**Fig. 3:**
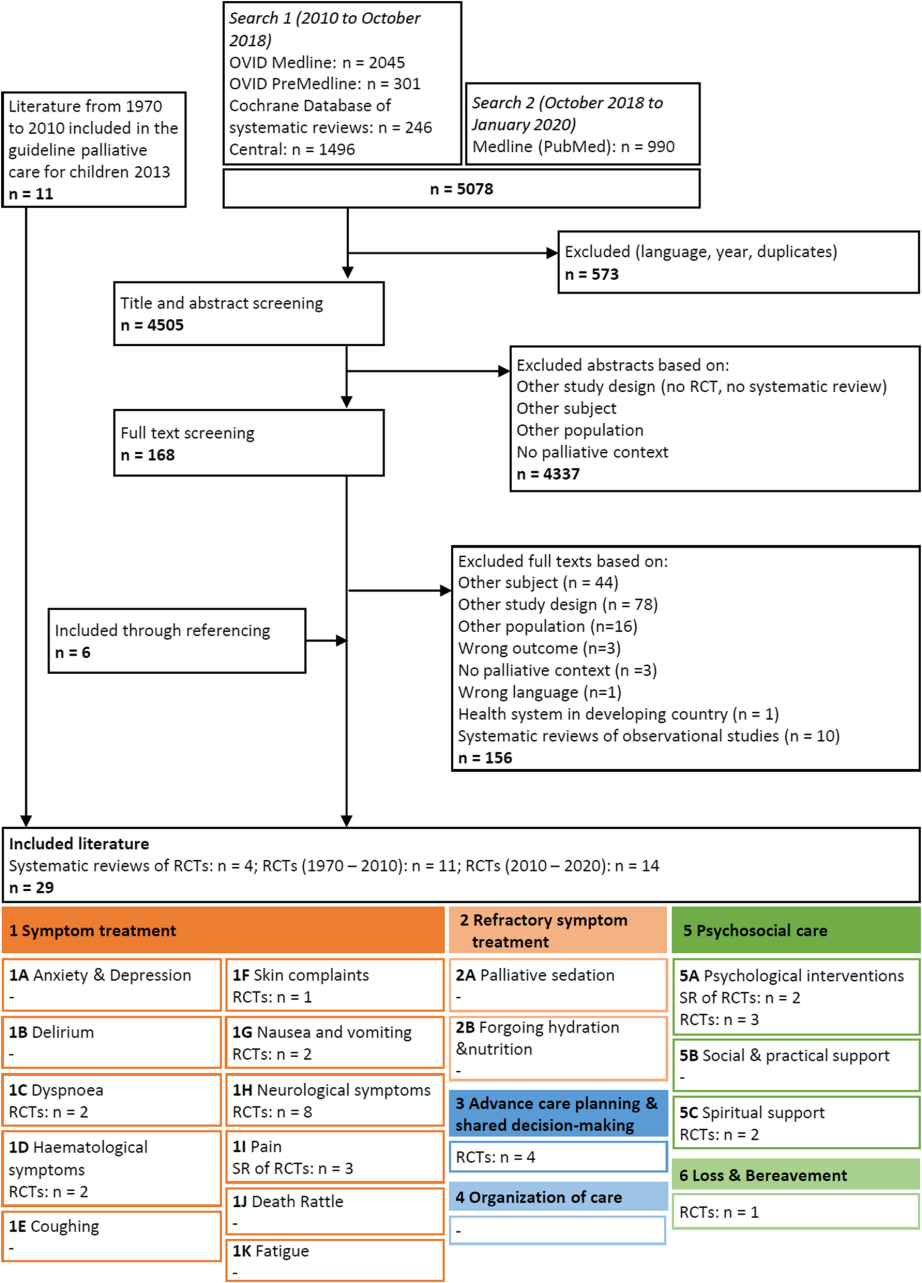
Flowchart of the selection process of quantitative studies

A total of 29 studies (25 RCTs and 4 SRs of RCTs) were eligible for inclusion. This included 11 RCTs that were identified in the previous CPG of 2013 and 18 newly published studies (Appendix J). We subsequently categorized all 29 citations according to topic. Then, we distributed the citations among the different WGs. Figure 3 shows a flow diagram of the study selection process.

### Identification of qualitative studies

For the formulated clinical question on barriers and facilitators of advance care planning and shared decision-making of WG3, the updated systematic literature search yielded 1232 eligible studies. A total of 1147 citations were excluded based on title/abstract and 85 citations were included for full text screening. Main reasons for exclusion of full texts were wrong study subject (no advance care planning or shared decision-making), wrong study design, or wrong study outcome (no barriers and facilitators).

**Fig. 4:**
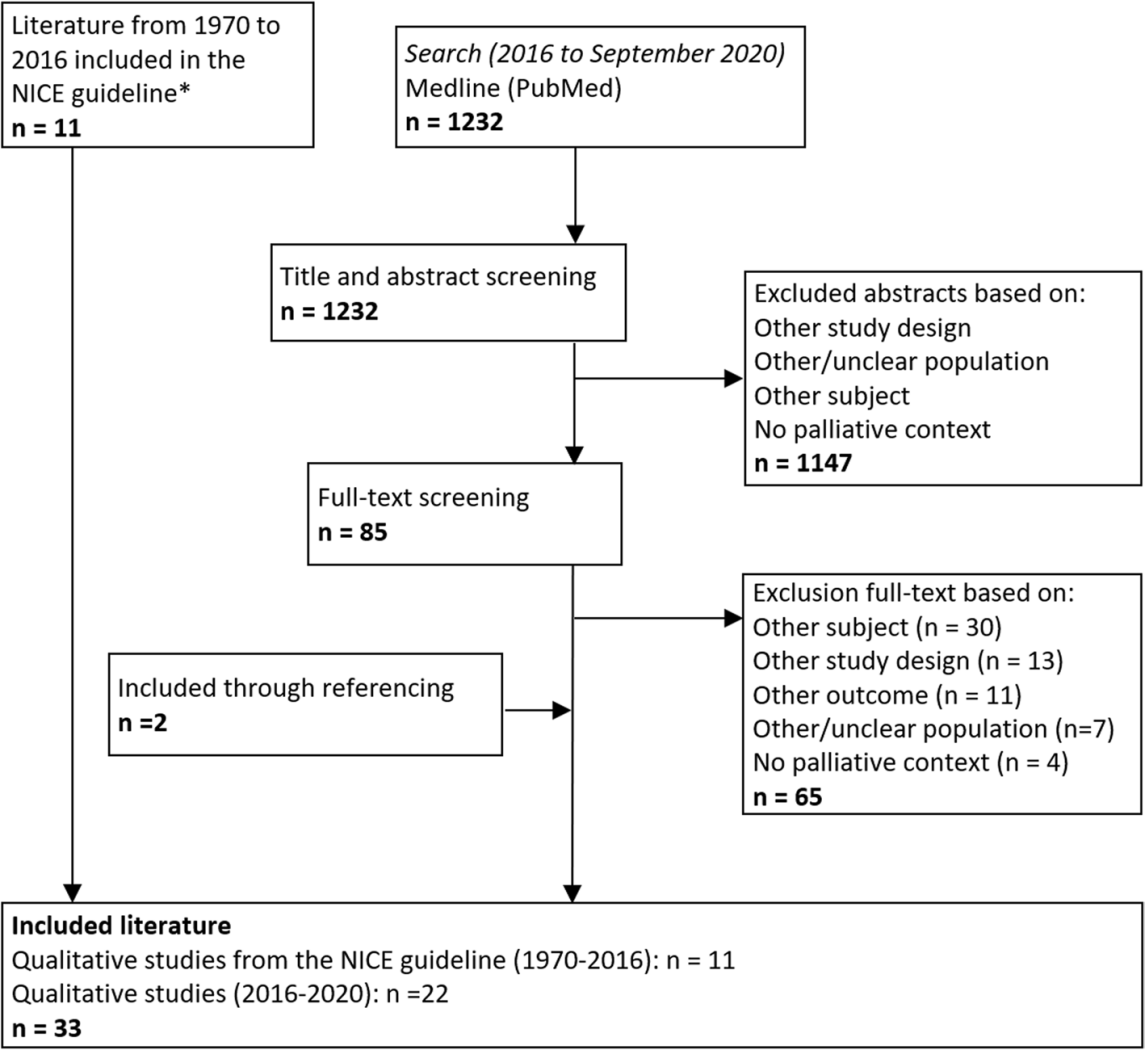
Flowchart of the selection process of qualitative studies *We only used the conclusions of evidence from the 11 identified studies in the search of the NICE guideline

A total of 33 studies on barriers and facilitators of advance care planning and shared decision-making were included. This included 22 newly published qualitative studies and 11 qualitative studies that were identified in the search of the NICE-guideline ‘End of life care for infants, children and young people with life-limiting conditions (2016)’ [12] (Appendix J). We used the conclusions of evidence of the 11 included studies in the NICE guideline and integrated these in our conclusions of evidence. Figure 4 shows a flow diagram of the selection process.

### Identification of additional literature

The search for guidelines identified 378 potential CPGs. In total, we included 29 CPGs of which 6 were paediatric palliative care CPGs, 11 were general paediatric CPGs and 12 were adult palliative care CPGs (Appendix J). Moreover, we included two textbooks on paediatric palliative care.

In addition, we included 10 SRs of observational, qualitative, or mixed-method studies on bereavement intervention components and features of communication strategies.

### Evidence base

Table 2 gives an overview of the selected studies from the systematic literature searches and selected additional literature per WG and clinical question. The systematic literature searches identified studies for 14 out of 38 formulated clinical questions, meaning that for 24 clinical questions the systematic literature searches identified no evidence. The number of identified studies from the systematic literature searches differed per WG and clinical question. Moreover, we were able to select additional literature, namely guidelines, textbooks, or SRs of observational, qualitative, or mixed-method studies for almost every clinical question.

**Table 2:** Selected studies per working group and clinical question

Topic/clinical question^a^	Selected studies from systematic literature searches	Selected additional literature
**WG1 Symptom treatment**		
1 Anxiety and depression: Non pharmacological interventions	-	4 guidelines [18–21]
2 Anxiety and depression: Pharmacological interventions	-	1 textbook [22], 4 guidelines [18–21]
3 Delirium: Non pharmacological interventions	-	2 guidelines [12, 23]
4 Delirium: Pharmacological interventions		
5 Dyspnoea: Non pharmacological interventions	2 RCTs [24, 25]	2 guidelines [20, 26]
6 Dyspnoea: Pharmacological interventions for dyspnoea	-	
7 Haematological symptoms: Pharmacological interventions for anaemia	2 RCTs [27, 28]	1 textbook [29], 2 guidelines [30, 31]
8 Haematological symptoms: Pharmacological interventions for thrombocytopenia	-	2 guidelines [30, 32]
9 Haematological symptoms: Pharmacological interventions for haemorrhages	-	-
10 Haematological symptoms: Pharmacological interventions for thrombosis	-	-
11 Coughing: Non pharmacological interventions	-	1 guideline [33]
12 Coughing: Pharmacological interventions		
13 Skin complaints: Non pharmacological interventions (pressure ulcers and itching)	-	5 guidelines [34–38]
14 Skin complaints: Pharmacological interventions (pressure ulcers and itching)	1 RCT [39]	
15 Nausea vomiting: Non pharmacological interventions	1 RCT [40]	2 guidelines [41, 42]
16 Nausea vomiting: Pharmacological interventions	7 RCTs [43–49]	4 guidelines [20, 41, 42, 50]
17 Neurological symptoms: Non pharmacological interventions	-	4 guidelines [51–54]
18 Neurological symptoms: Pharmacological interventions	2 RCTs [55, 56]	5 guidelines [12, 51–54]
19 Pain: Non pharmacological interventions	1 SR of RCTs [57]	1 guideline [58]
20 Pain: Pharmacological interventions	2 SR of RCTs [59, 60]	1 guideline [12]
21 Death rattle: Non pharmacological interventions	-	2 guidelines [12, 61]
22 Death rattle: Pharmacological interventions		
23 Fatigue: Non pharmacological interventions	-	2 guidelines [62, 63]
24 Fatigue: Pharmacological interventions		1 guideline [62]
**WG2 Refractory symptom treatment**		
25 Palliative sedation	-	2 textbooks [22, 29], 1 guideline [64]
26 Palliative sedation in children with severe disabilities		-
27 Forgoing hydration and nutrition	-	2 textbooks [22, 29], 2 guidelines [12, 65]
**WG3 Advance care planning and shared decision-making**		
28 Advance care planning interventions	4 RCTs [66–69]	1 guideline [12]
29 Barriers and facilitators of advance care planning and shared-decision making	22 qualitative studies [70–91] ^b^	
**WG4 Organisation of care**		
30 Organisational interventions	-	1 guidelines [12]
**WG5 Psychosocial care**		
31 Psychological interventions for children	1 SR of RCTs [92], 2 RCTs [93, 94]	1 guideline [12]
32 Psychological interventions for parents, and family members	2 SRs of RCTs [57, 92], 1 RCT [95]	
33 Social and practical support for children, parents, and family members	-	
34 Cultural, spiritual, and religious support for children, parents, and family members	2 RCTs [96, 97]	
**WG6 Loss and bereavement**		
35 Bereavement care interventions for children, parents, and family members	1 RCT [98]	1 guideline [12]
36 Components of bereavement care interventions	-	10 SRs [99–108], 1 guideline [12]
37 Experiences and needs of parents and health care providers		
38 Features of communicative and affective strategies		

^a^Complete clinical questions can be found in Appendix D

^b^Conclusions of evidence from 11 studies on barriers and facilitators of ACP and shared decision making of the NICE guideline ‘End of life care for infants, children and young people with life-limiting conditions (2016)’ were used

## Discussion

Over the years, significant progress has been made in improving and integrating paediatric palliative care in the Netherlands [109]. The first Dutch CPG for paediatric palliative care contributed to the quality and organisation of palliative care for children with life-threatening and life-limiting conditions [109]. Several years after the development of the first Dutch CPG for paediatric palliative care, health care providers, parents, and other stakeholders expressed the need for more guidance on specific topics that were not covered in the first CPG (such as palliative sedation and forgoing hydration and nutrition). This, together with the need to review evidence and build a stronger evidence-base from scientific literature, inspired the revision of the Dutch CPG for paediatric palliative care.

In this paper, we provide a complete overview of our methodology to revise the Dutch CPG for paediatric palliative care and give a brief presentation of the identified evidence. By sharing our methodology, we hope to promote transparency in CPG development and support others in developing approaches which we used to deal with expected challenges (multiple topics, lack of evidence, improve applicability and implementation) and unexpected challenges (high workload).

First, we used an evidence-based approach to revise the Dutch CPG for paediatric palliative care. We expected this would be challenging, as we chose to include a total of 38 clinical questions covering 16 topics related to paediatric palliative care. Therefore, we developed an approach that prevented unmanageable amounts of work without compromising quality. We decided to update existing systematic literature searches and mainly focus on high quality evidence. For all clinical questions on paediatric palliative care interventions, we updated the systematic literature search that was used in the first CPG to identify quantitative evidence (RCTs/CCTs and SRs of RCTs) on all paediatric palliative care interventions. For the clinical question on barriers and facilitators of advance care planning and shared decision-making, we updated the systematic literature search to identify qualitative evidence. This search was originally developed by the NICE guideline ‘End of life care for infants, children and young people with life-limiting conditions: planning and management’ [12]. By re-using evidence from previous systematic literature searches and combine it with new identified evidence, we were able to formulate a large set of recommendations.

Additionally, we developed an approach to deal with the lack of evidence. As the systematic literature search that was conducted for the first CPG identified little evidence, we expected a lack of evidence for the update as well. To deal with this challenge, we decided to search for additional literature sources to base recommendations upon. We used textbooks on paediatric palliative care, and guidelines on paediatric palliative care, general paediatrics, and adult paediatric palliative care and SRs of observational, qualitative, or mixed-method studies. Additional literature was selected according to its relevance.

Moreover, as stakeholders expressed a need for more guidance on certain topics related to paediatric palliative care, we created an approach to further improve applicability as well as implementation of this guideline. Therefore, we decided to not only focus on physical aspects (symptom relief), decision-making, and organisation of care but also on other topics such as advance care planning, psychological, social, and spiritual support, and loss and bereavement. These topics are increasingly recognised as important within paediatric palliative care [109]. Furthermore, the selected topics are based on priorities of health care providers and parents. As a result, we believe this increases the likelihood that the recommendations of this guideline will be applied in practice.

To further improve dissemination and implementation of the guideline, we collaborated with many stakeholders including health care providers from multiple disciplines and parent representatives. It has been shown that patient values improve quality of CPGs and is invaluable [110]. Therefore, parent representatives have been involved in different ways throughout the entire guideline process, which ensures representation of patients and their families. Additionally, this guideline is approved by all relevant professional and patient associations in the Netherlands, meaning that these associations consider the CPG as a standard for provision of paediatric palliative care. Ultimately, we believe that this approach will lead to increased dissemination and implementation of the revised CPG among health care providers, parents, and children.

The revision of this guideline entailed a significantly greater amount of effort than we initially anticipated due to time-intensive tasks such as the selection and appraisal of evidence, and instruction and motivation of all guideline panel members. We appointed one researcher who coordinated the entire guideline development process on a fulltime basis for more than three years. This approach turned out to be very beneficial as it improved collaboration between all guideline panel members and contributed to a smooth process as issues were timely addressed. We therefore highly recommend others to adopt this approach, especially in situations where lack of time and resources might be an issue.

Unfortunately, despite our efforts to deal with (un)expected challenges, we found that there are still many knowledge gaps in paediatric palliative care for children. We identified no evidence for 24 out of 38 clinical questions, mainly including questions on (refractory) symptoms. It should be noted that we included evidence from 1970 to 2020. Therefore, it is plausible that we missed some recently published evidence. However, based on the little studies we found in a large time frame; it is plausible we miss only a small number of studies that most likely will not have a direct influence on our identified knowledge gaps. As a result, we emphasize the need for more high-quality research on paediatric palliative care interventions to further improve quality of care.

Furthermore, we acknowledge that we describe the methodology of a national CPG for paediatric palliative care. Although we use international evidence, our recommendations will be largely based on national clinical expertise and patient experiences due to identified knowledge gaps. Based on our previous experiences, we believe that the targeted recommendations we will provide in this guideline, will positively influence the further integration of paediatric palliative care in the Netherlands. Moreover, as we used international evidence, we believe that a large proportion of provided recommendations, except recommendations that are specific for the Dutch context (organisation of care), will be applicable to other contexts and can be of great added value. Especially, since we provide a comprehensive set of recommendations for all children and their families in need of palliative care from beginning of diagnosis till after the-end-life. However, country-specific factors such as availability of (non)pharmacological interventions, infrastructure, financial resources, and cultural backgrounds, should always be carefully considered before applying any recommendations in other contexts.

## Conclusions

We developed a transparent evidence-based methodology for the revision of the Dutch CPG for paediatric palliative care. Within this methodology, we developed approaches to deal with lack of evidence and improve applicability of the guideline by incorporating patient and family values and experiences throughout the entire guideline process. Our methodology combines existing evidence from scientific literature, additional literature, expert knowledge, and perspectives of patients and their families to formulate recommendations on all domains of paediatric palliative care (medical, psychological, social, and spiritual care). By using this methodology, we aim to develop the most comprehensive evidence-based guideline in paediatric palliative care.

### Supplementary Information

**Additional file 1:  Appendix (A).** Survey among health care professionals to select guideline topics. **Appendix (B).** Survey among patient representatives and parents to select guideline topics. **Appendix (C).** Paediatric palliative care guideline panel; **Appendix (D).** Working structure for guideline development; **Appendix (E).** Guideline development process; **Appendix (F).** Clinical questions; **Appendix (G).** Search strategies; **Appendix (H).** Inclusion criteria; **Appendix (I).** Criteria for appraisal of evidence and strength of recommendations; **Appendix (J).** Results: identified evidence and selected additional literature.

## Data Availability

All data analysed during this study is included in this published article and Additional information file 1.

## Abbreviations

CASP: Critical Appraisal Skills Programme
CPG: Clinical Practice Guideline
CCT: Controlled Clinical Trial
CENTRAL: Cochrane Central Register of Controlled Trials
GRADE: Grading Recommendation Assessment Development and Evaluation
GRADE CERQual: Grading Recommendation Assessment Development and Evaluation Confidence in the Evidence from Reviews of Qualitative research
iPOG: International Paediatric Oncology Guidelines in supportive care
MEDLINE: Medical Literature Analysis and Retrieval System Online
NICE: National Institute of Health Care Excellence
PICOS: Patient, Intervention, Comparison, Outcomes, Study design
RCT: Randomized Controlled Trial
SR: Systematic Review
WG: Working Group
WG1: Working group 1: Symptom treatment
WG2: Working group 2: Refractory symptom treatment
WG3: Working group 3: Advance care planning and shared decision making
WG4: Working group 4: Organisation of care
WG5: Working group 5: Psychosocial care
WG6: Working group 6: Loss and bereavement
WHO: World Health Organization
