# Supplementary material for: Palliative care for children: methodology for the development of a national clinical practice guideline
Source: BMC Palliat Care. 2023 Dec 1;22:193. doi: 10.1186/s12904-023-01293-3 (PMC10691037; doi:10.1186/s12904-023-01293-3)
Supplement: Supplementary file 1 — Additional file 1: Appendix (A). Survey among health care professionals to select guideline topics. Appendix (B). Survey among patient representatives and parents to select guideline topics. Appendix (C). Paediatric palliative care guideline panel; Appendix (D). Working structure for guideline development; Appendix (E). Guideline development process; Appendix (F). Clinical questions; Appendix (G). Search strategies; Appendix (H). Inclusion criteria; Appendix (I). Criteria for appraisal of evidence and strength of recommendations; Appendix (J). Results: identified evidence and selected additional literature. [file 12904_2023_1293_MOESM1_ESM.docx]

**APPENDICES**

Palliative care for children: Methodology for the development of a national clinical practice guideline

**Content**

[Appendix A. Survey among health care professionals to select guideline topics 2](#_Toc138164374)

[Appendix B. Survey among patient representatives and parents to select guideline topics 6](#_Toc138164375)

[Appendix C. Paediatric palliative care guideline development panel 15](#_Toc138164376)

[Appendix D. Working structure for guideline development 20](#_Toc138164377)

[Appendix E. Guideline development process 21](#_Toc138164378)

[Appendix F. Clinical questions 22](#_Toc138164379)

[Appendix G. Search strategies 26](#_Toc138164380)

[Appendix H. Inclusion criteria 30](#_Toc138164381)

[Appendix I. Criteria for appraisal of evidence and strength of recommendations. 33](#_Toc138164382)

[Appendix J. Results: identified evidence and selected additional literature 34](#_Toc138164383)

# Appendix A. Survey among health care professionals to select guideline topics

**General questions**

1. What is your area of expertise?

Paediatrician

Physical therapist

Spiritual caretaker / chaplain

General practitioner

Social worker

Medical specialist (other than paediatrician)

Psychologist

Rehabilitation doctor

Home nurse

Nurse

Nurse practitioner

Member of paediatric palliative care team

Member of a paediatric palliative care network

Other: ………………………………….

1. How do you respond?

On personal title

On behalf of (institution, professional association or scientific association)

1. Within which healthcare setting are you working?

Academic hospital

General hospital

Hospice

General practitioners’ practice

Institution for psychosocial care

Home care

Other: ………………………………

1. On average, how many children in the palliative phase do you attend to per year?

None

1 to 5

5 to 10

More than 10

**Issues related to diagnostics in paediatric palliative care.**

1. For which topics regarding diagnostics in paediatric palliative care do you experience issues?

The following topics on diagnostics in paediatric palliative care have been selected.

- Please select the five most important topics for which you experience issues. For each of these five topics, indicate the priority on a scale of extremely important to least important.
- Each checkbox can only be ticked once.
- You can only pick five topics; the remaining topics remain empty. If your topic is not included, please indicate this at the bottom.

|  | Extremely important | Very important | Fairly important | Less important | Least important |
| --- | --- | --- | --- | --- | --- |
| Anxiety and depression |  |  |  |  |  |
| Epileptic seizures |  |  |  |  |  |
| Haematological symptoms |  |  |  |  |  |
| Coughing and death rattle |  |  |  |  |  |
| Skin complaints |  |  |  |  |  |
| Nausea and vomiting |  |  |  |  |  |
| Neurological symptoms and loss of neurological function |  |  |  |  |  |
| Pain |  |  |  |  |  |
| Psychosocial problems |  |  |  |  |  |
| Spasticity |  |  |  |  |  |
| Fatigue |  |  |  |  |  |

Would you like to comment, or do you want to share another topic related to diagnostics in paediatric palliative care with the guideline development panel?

(Please list the topic first and then state your comment or issue).

**Issues related to treatment in paediatric palliative care**

1. For which topics regarding treatment in paediatric palliative care do you experience issues?

The following topics on treatment in paediatric palliative care have been selected.

- Please select the five most important topics for which you experience issues. For each of these five topics, indicate the priority on a scale of extremely important to least important.
- Each checkbox can only be ticked once.
- You can only pick five topics; the remaining topics remain empty. If your topic is not included, please indicate this at the bottom.

|  | Extremely important | Very important | Fairly important | Less important | Least important |
| --- | --- | --- | --- | --- | --- |
| Anxiety and depression |  |  |  |  |  |
| Epileptic seizures |  |  |  |  |  |
| Haematological symptoms |  |  |  |  |  |
| Coughing and death rattle |  |  |  |  |  |
| Skin complaints |  |  |  |  |  |
| Nausea and vomiting |  |  |  |  |  |
| Neurological symptoms and loss of neurological function |  |  |  |  |  |
| Pain |  |  |  |  |  |
| Psychosocial problems |  |  |  |  |  |
| Spasticity |  |  |  |  |  |
| Fatigue |  |  |  |  |  |

Would you like to comment, or do you want to share another topic related to treatment in paediatric palliative care with the guideline development panel?

(Please list the topic first and then state your comment or issue).

**Issues related to decision-making in paediatric palliative care**

1. For which topics regarding decision-making in paediatric palliative care do you experience issues?

Please prioritise the topics below by indicating how important you consider this topic to be. If your topic is not included, please indicate this at the bottom.

|  | Extremely important | Very important | Fairly important | Less important | Least important |
| --- | --- | --- | --- | --- | --- |
| General aspects of decision-making |  |  |  |  |  |
| Decision-making on symptom treatment |  |  |  |  |  |
| Organisational aspects of decision-making |  |  |  |  |  |

Would you like to comment, or do you want to share another topic related to decision-making in paediatric palliative care with the guideline development panel?

(Please list the topic first and then state your comment or issue).

**Issues related to organisation of paediatric palliative care**

1. For which topics regarding organisation of paediatric palliative care do you experience issues?

Please prioritise the topics below by indicating how important you consider this topic to be. If your topic is not included, please indicate this at the bottom of the list.

|  | Extremely important | Very important | Fairly important | Less important | Least important |
| --- | --- | --- | --- | --- | --- |
| Coordination of care |  |  |  |  |  |
| Care plan |  |  |  |  |  |
| Collaborative models in paediatric palliative care |  |  |  |  |  |

Would you like to comment, or do you want to share another topic related to organisation of paediatric palliative care with the guideline development panel?

(Please list the topic first and then state your comment or issue).

**Comments**

1. Do you have any advice or comments regarding the revision of the guideline for paediatric palliative care?

10. Do you have any comments on the survey?

# Appendix B. Survey among patient representatives and parents to select guideline topics

**Personal information**

1. Are you a parent/caregiver of a child with a life-limiting or life-threatening condition?

Yes

No

1. What is your child’s date of birth?
2. In what year was your child diagnosed?
3. What is the gender of your child with this diagnosis?

Female

Male

Gender neutral

I’d rather not say

1. What is your gender?

Female

Male

1. Do you have experience with loss and bereavement care because your child has unfortunately passed away?

Yes

No

1. In what year did your child pass away?

**Theme 1: Psychosocial care, loss, and bereavement**

1. Please indicate for each topic related to theme 1, how important this topic is for you and your family.

|  | Not important | Fairly important | Neutral | Important | Very important | Don’t know | NA |
| --- | --- | --- | --- | --- | --- | --- | --- |
| 1. Communication with the child on non-medical aspects |  |  |  |  |  |  |  |
| 1. Communication with the family on non-medical aspects |  |  |  |  |  |  |  |
| 1. Attention for social and emotional consequences for the child |  |  |  |  |  |  |  |
| 1. Attention for social and emotional consequences for the parents/caregivers |  |  |  |  |  |  |  |
| 1. Attention for social and emotional consequences for brothers and sisters |  |  |  |  |  |  |  |
| 1. Attention for balance between care, work, and family life |  |  |  |  |  |  |  |
| 1. Attention for the child’s development |  |  |  |  |  |  |  |
| 1. Attention for social activities of the child |  |  |  |  |  |  |  |
| 1. Attention for the child’s participation in education and day care |  |  |  |  |  |  |  |
| 1. Attention for the safety of the child |  |  |  |  |  |  |  |
| 1. Attention for empathy, appreciation, respect, and privacy towards child and family |  |  |  |  |  |  |  |
| 1. Attention for cultural background, religion, and spirituality |  |  |  |  |  |  |  |
| 1. Social and emotional support for child and family |  |  |  |  |  |  |  |
| 1. Grief counselling for the family after death |  |  |  |  |  |  |  |

1. Priority of the topics in theme 1.

Now we ask you to prioritize the topics related to theme 1 that you have just assessed.

- Which five topics from this theme do you see as the most important topic?
- You may choose a maximum of five topics. It is also possible to choose less than five topics, if you do not consider more topics important. Click on a topic on the left-hand side of the screen to move it to your priority list.
- The topic you find most important will appear at the top right of the screen. You can move a topic up or down using the blue arrow. Use the blue cross behind the selected topic to remove it from the list.

a. Communication with the child on non-medical aspects

b. Communication with the family on non-medical aspects

c. Attention for social and emotional consequences for the child

d. Attention for social and emotional consequences for the parents/caregivers

e. Attention for social and emotional consequences for brothers and sisters

f. Attention for balance between care, work, and family life

g. Attention for the child’s development

h. Attention for social activities of the child

i. Attention for the child’s participation in education and day care

j. Attention for the safety of the child

k. Attention for empathy, appreciation, respect, and privacy towards child and family

l. Attention for cultural background, religion, and spirituality

m. Social and emotional support for child and family

n. Grief counselling for the family after death

1. Have you missed any topics related to theme 1 that should be addressed? If so, please add them below.
2. Is there any specific question related to theme 1, you would like to have answered in the guideline?

**Theme 2: Advance Care Planning**

1. Please indicate for each topic, how important this topic is for you and your family.

|  | Not important | Fairly important | Neutral | Important | Very important | Don’t know | NA |
| --- | --- | --- | --- | --- | --- | --- | --- |
| 1. Being ready to start talking about future care and treatment as a family |  |  |  |  |  |  |  |
| 1. Attention from health care professionals to check if you are ready to talk about future care and treatment as a family |  |  |  |  |  |  |  |
| 1. Support to the family to talk about future care and treatment |  |  |  |  |  |  |  |
| 1. Timely discussion about care and treatment with parents initiated by the healthcare professional |  |  |  |  |  |  |  |
| 1. Timely discussion about care and treatment with child (if old enough and able to) initiated by the health care professional |  |  |  |  |  |  |  |

1. Priority of the topics in theme 2.

Now we ask you to prioritize the topics related to theme 2 that you have just assessed.

- Which five topics from this theme do you see as the most important topic?
- You may choose a maximum of five topics. It is also possible to choose less than five topics, if you do not consider more topics important. Click on a topic on the left-hand side of the screen to move it to your priority list.
- The topic you find most important will appear at the top right of the screen. You can move a topic up or down using the blue arrow. Use the blue cross behind the selected topic to remove it from the list.

a. Being ready to start talking about future care and treatment as a family

b. Attention from health care professionals to check if you are ready to talk about future care and treatment as a family

c. Support to the family to talk about future care and treatment

d. Timely discussion about care and treatment with parents initiated by the healthcare professional

e. Timely discussion about care and treatment with child (if old enough and able to) initiated by the health care professional

1. Have you missed any topics related to theme 2 that should be addressed? If so, please add them below.
2. Is there any specific question related to theme 2, you would like to have answered in the guideline?

**Theme 3: Organisation of care**

1. Please indicate for each topic, how important this topic is for you and your family.

|  | Not important | Fairly important | Neutral | Important | Very important | Don’t know | NA |
| --- | --- | --- | --- | --- | --- | --- | --- |
| 1. Transition of care from hospital to home |  |  |  |  |  |  |  |
| 1. Mapping wishes and needs of child and family on spirituality and the four child life domains: medical, psychosocial, safety and development |  |  |  |  |  |  |  |
| 1. Social and emotional impact on child and family is part of the treatment plan |  |  |  |  |  |  |  |
| 1. Self-management in care and treatment |  |  |  |  |  |  |  |
| 1. Health care professionals have knowledge on the social services available for child and family |  |  |  |  |  |  |  |
| 1. Indication with attention to spirituality and the four child life domains: medical, psychosocial, safety and development |  |  |  |  |  |  |  |
| 1. Indication process |  |  |  |  |  |  |  |
| 1. Aligning care needs with the indication |  |  |  |  |  |  |  |
| 1. Availability of suitable care |  |  |  |  |  |  |  |
| 1. Appropriate provision of respite care and lodging facilities |  |  |  |  |  |  |  |
| 1. Organisation of suitable care |  |  |  |  |  |  |  |
| 1. Legislation and regulation |  |  |  |  |  |  |  |
| 1. Shared decision-making on organisation of care |  |  |  |  |  |  |  |
| 1. Clarity on who is the head practitioner |  |  |  |  |  |  |  |
| 1. Collaboration between the different (health care) professionals |  |  |  |  |  |  |  |
| 1. Alignment of information between the different (health care) professionals |  |  |  |  |  |  |  |
| 1. Coordination of care (case management) |  |  |  |  |  |  |  |
| 1. In addition to focusing on the medical domain, also focus on social, safe and development in approach to care and care plan (integral/holistic) |  |  |  |  |  |  |  |
| 1. Shared-decision making at the end-of-life |  |  |  |  |  |  |  |
| 1. Digital individual care used by all health care professionals |  |  |  |  |  |  |  |
| 1. Transition to adult care |  |  |  |  |  |  |  |

1. Priority of the topics in theme 3.

Now we ask you to prioritize the topics related to theme 3 that you have just assessed.

- Which five topics from this theme do you see as the most important topic?
- You may choose a maximum of five topics. It is also possible to choose less than five topics, if you do not consider more topics important. Click on a topic on the left-hand side of the screen to move it to your priority list.
- The topic you find most important will appear at the top right of the screen. You can move a topic up or down using the blue arrow. Use the blue cross behind the selected topic to remove it from the list.

a. Transition of care from hospital to home

b. Mapping wishes and needs of child and family on spirituality and the four child life domains: medical, psychosocial, safety and development

c. Social and emotional impact on child and family is part of the treatment plan

d. Self-management in care and treatment

e. Health care professionals have knowledge on the social services available for child and family

f. Indication with attention to spirituality and the four child life domains: medical, psychosocial, safety and development

g. Indication process

h. Aligning care needs with the indication

i. Availability of suitable care

j. Appropriate provision of respite care and lodging facilities

k. Organisation of suitable care

l. Legislation and regulation

m. Shared decision-making on organisation of care

n. Clarity on who is the head practitioner

o. Collaboration between the different (health care) professionals

p. Alignment of information between the different (health care) professionals

q. Coordination of care (case management)

r. In addition to focusing on the medical domain, also focus on social, safe and development in approach to care and care plan (integral/holistic)

s. Shared-decision making at the end-of-life

t. Digital individual care used by all health care professionals

u. Transition to adult care

1. Have you missed any topics related to theme 3, that should be addressed? If so, please add them below.
2. Is there any specific question related to theme 3, you would like to have answered in the guideline?

**Theme 4: Symptoms: diagnostics and treatment**

1. Please indicate for each topic, how important this topic is for you and your family.

|  | Not important | Fairly important | Neutral | Important | Very important | Don’t know | NA |
| --- | --- | --- | --- | --- | --- | --- | --- |
| 1. Deciding together with parents when choosing medical treatment |  |  |  |  |  |  |  |
| 1. Deciding together with child (if old enough and able to) when choosing medical treatment |  |  |  |  |  |  |  |
| 1. Attention for the rights of the child to be informed and to take part in decision making |  |  |  |  |  |  |  |
| 1. Attention for the child's quality of life when choosing care and treatments |  |  |  |  |  |  |  |
| 1. Attention for the prevention of anxiety, stress and pain when choosing and implementing care and treatment |  |  |  |  |  |  |  |
| 1. Anxiety and depression in the child |  |  |  |  |  |  |  |
| 1. Delirium |  |  |  |  |  |  |  |
| 1. Dyspnoea (feeling of not getting enough air) |  |  |  |  |  |  |  |
| 1. Haematological symptoms such as anaemia, bleeding, and thrombosis |  |  |  |  |  |  |  |
| 1. Coughing |  |  |  |  |  |  |  |
| 1. Skin complaints |  |  |  |  |  |  |  |
| 1. Nausea and vomiting |  |  |  |  |  |  |  |
| 1. Fatigue |  |  |  |  |  |  |  |
| 1. Death rattle (this occurs because mucus collects in the pharynx. It is a common symptom in the last stage of life) |  |  |  |  |  |  |  |
| 1. Neurological symptoms (epileptic seizures or spasticity) |  |  |  |  |  |  |  |
| 1. Pain |  |  |  |  |  |  |  |
| 1. Refractory symptoms (difficult to treat symptoms at the end-of-life) |  |  |  |  |  |  |  |

1. Priority of the topics in theme 4.

Now we ask you to prioritize the topics related to theme 4 that you have just assessed.

- Which five topics from this theme do you see as the most important topic?
- You may choose a maximum of five topics. It is also possible to choose less than five topics, if you do not consider more topics important. Click on a topic on the left-hand side of the screen to move it to your priority list.
- The topic you find most important will appear at the top right of the screen. You can move a topic up or down using the blue arrow. Use the blue cross behind the selected topic to remove it from the list.

a. Deciding together with parents when choosing medical treatment

b. Deciding together with child (if old enough and able to) when choosing medical treatment

c. Attention for the rights of the child to be informed and to take part in decision making

d. Attention for the child's quality of life when choosing care and treatments

e. Attention for the prevention of anxiety, stress and pain when choosing and implementing care and treatment

f. Anxiety and depression in the child

g. Delirium

h. Dyspnoea (feeling of not getting enough air)

i. Haematological symptoms such as anaemia, bleeding, and thrombosis

j. Coughing

k. Skin complaints

l. Nausea and vomiting

m. Fatigue

n. Death rattle (this occurs because mucus collects in the pharynx. It is a common symptom in the last stage of life)

o. Neurological symptoms (epileptic seizures or spasticity)

p. Pain

q. Refractory symptoms (difficult to treat symptoms at the end-of-life)

1. Have you missed any topics related to theme 4 that should be addressed? If so, please add them below.
2. Is there any specific question related to theme 4, you would like to have answered in the guideline?
3. Which of the mentioned themes do you find most important?

Which theme is most important for you and your family. Please indicate the desired order. The top theme is the most important theme for you and the bottom one is the least important.

Theme 1: Psychosocial care, loss, and bereavement

Theme 2: Advance Care Planning

Theme 3: Organisation of care

Theme 4: Symptoms: diagnostics and treatment

1. Is there anything else you would like to share with us?
2. Would you like to read along with the text that is written by the guideline development panel?

Yes

No

# Appendix C. Paediatric palliative care guideline development panel

**Expert panel**

Core group members

| Name | Role | Area of expertise |
| --- | --- | --- |
| Erna Michiels | Chair | Paediatric oncology, paediatric palliative care |
| Eduard Verhagen | Co-Chair | Paediatrics, paediatric palliative care |
| Kim van Teunenbroek | Coordinator | Guideline development (PhD candidate) |
| Leontien Kremer | Advisor | Guideline development, paediatrics, paediatric oncology |
| Renée Mulder | Advisor | Guideline development, Paediatric Oncology |
| Hester Rippen | Patient representative (Stichting Kind & Ziekenhuis) | |
| Johannes Verheijden | Patient representative (Dutch Knowledge Centre for Children’s Palliative Care) | |
| Brigitt Borggreve | Process support (advisor palliative care IKNL) | |
| Fleur Godrie | Process support (advisor palliative care IKNL) | |
| Inge van Trigt | Process support (advisor palliative care IKNL) | |
| Francis Essers | Process support (secretary IKNL) | |

Working group members

| Name | Profession | Working group - Role |
| --- | --- | --- |
| **WG chairs** | | |
| Jeffry Looijestijn | Health care psychologist | Anxiety and Depression (WG 1A) – Chair  Psychological interventions (WG 5A) – WG member |
| Jolanda Schieving | Paediatric neurologist | Delirium (WG 1B) - Chair  Neurological symptoms (WG 1H) - Chair |
| Carin Delsman-van Gelder | Paediatrician in training | Delirium (WG 1B) - Chair  Neurological symptoms (WG 1H) - Chair  Refractory symptom treatment (WG 2) – WG member  Advance care planning (ACP) and shared decision-making (SDM) (WG3) – WG member |
| Marinka de Groot | Nurse practitioner specialized in paediatric palliative care | Dyspnoea (WG 1C) - Chair  Death rattle (WG 1J) – WG member  Refractory symptom treatment (WG 2) – WG member |
| Katja Heitink-Polle | Paediatric haematologist and oncologist | Haematological symptoms (WG 1D) - Chair |
| Inge Ahout | Paediatrician | Coughing (WG 1E) - Chair  Death rattle (WG 1J) - Chair  Nausea and vomiting (WG 1G) – WG member |
| Annemie Galimont | Dermatologist | Skin complaints (WG 1F) - Chair |
| Karin Bindels-de Heus | Paediatrician - genetic developmental disorders | Nausea and vomiting (WG 1G) - Chair  Advance care planning (ACP) and shared decision-making (SDM) (WG3) - Reviewer  Social and practical support (WG 5B) - Reviewer |
| Maarten Mensink | Paediatric anaesthesiologist | Pain (WG 1I) - Chair |
| Selma Mulder | Child occupational therapist | Fatigue (WG 1K) - Chair  Neurological symptoms (WG 1H) - Reviewer |
| Netteke Schouten-van Meeteren | Paediatrician, paediatric oncologist | Refractory symptoms treatment (WG 2) - Chair |
| Marijke Kars | Associate professor, paediatric nurse | ACP and SDM (WG 3) – Chair  Spiritual support (WG 5C) - Reviewer  Loss and Bereavement (WG 6) – Co-Chair |
| Jurrianne Fahner | Paediatrician, researcher Paediatric Palliative Care | ACP and SDM (WG 3)– Co-Chair |
| Mirjam de Vos-Broerse | Orthopedagogue, senior investigator paediatric palliative care | ACP and SDM (WG 3)– Co-Chair |
| Petra Honig-Mazer | Psychotherapist | Psychological interventions (WG 5A) – Chair  Social and Practical Support (WG 5B) - Chair |
| Nette Falkenburg | Spiritual caregiver | Spiritual Support (WG 5C) – Chair  Loss and Bereavement (WG 6) - Reviewer |
| Eline Kochen | Psychologist, PhD candidate loss and bereavement | Loss and Bereavement (WG 6) – Chair |
| Karolien Kisman | General practitioner, physician palliative care | Organisation of care (WG 4) - Chair |
| **WG members** | | |
| Esther van den Bergh | Clinical psychologist | Anxiety and Depression (WG 1A) – WG member  Fatigue (WG 1K) – WG member  Loss and Bereavement (WG 6) – WG member |
| Kim van der Schoot | Health care psychologist | Anxiety and Depression (WG 1A) – WG member |
| Hennie Knoester | Paediatrician, paediatric intensivist | Delirium (WG 1B) – WG member  Psychological interventions (WG 5A) – Reviewer  Social and Practical Support (WG 5B) – Reviewer |
| Mariska Nieuweboer | Paediatric oncology nurse | Delirium (WG 1B) – WG member  Dyspnoea (WG 1C) – WG member  Haematological symptoms (WG 1D) – WG member  Pain (WG 1I) – WG member |
| Willemien de Weerd | Paediatrician | Dyspnoea (WG 1C) – WG member  Haematological symptoms (WG 1D) – WG member  Skin complaints - Reviewer |
| Saskia Gischler | Paediatrician, intensivist | Haematological symptoms (WG 1D) – WG member  Skin complaints (WG 1F) - Reviewer  Refractory symptom treatment (WG 2) – WG member |
| Arno Colenbrander | Paediatrician | Coughing (WG 1E) – WG member  Death rattle (WG 1J) – WG member |
| Govert Brinkhorst | Paediatrician, pulmonologist | Coughing (WG 1E) – WG member  Death rattle (WG 1J) – WG member |
| Leo van Vlimmeren | Child physiotherapist | Coughing (WG 1E) – WG member  Death rattle (WG 1J) – WG member |
| Suzanne Pasmans | (Child)dermatologist, immunologist | Skin Complaints (WG 1F) – WG member |
| Barbara de Koning | Paediatrician, gastroenterologist | Nausea and vomiting (WG 1G) – WG member |
| Irma Rigter | Hospital pharmacist | Nausea and vomiting (WG 1G) – WG member |
| Christel Rohrich | Paediatric rehabilitation specialist | Neurological symptoms (WG 1H) – WG member  Fatigue (WG 1K) - Reviewer  Organisation of care (WG 4) – Reviewer |
| Karin Geleijns | Neurologist | Neurological symptoms (WG 1H) – WG member |
| Ellen Siegers-Bennink | Pain consultant | Pain (WG 1I) – WG member |
| Tanneke Snijders-Groenedijk | Medical social care provider | Fatigue (WG 1K) – WG member |
| Mattijs Alsem | Paediatric rehabilitation specialist | Fatigue (WG 1K) – WG member  ACP and SDM (WG 3) – Reviewer  Organisation of care (WG 4) – WG member  Psychological interventions (WG 5A) – Reviewer  Social and Practical Support (WG 5B) – Reviewer |
| Liesbeth Ruijgrok | Hospital pharmacist | Refractory symptom treatment (WG 2) – WG member |
| Laurent Favié | Hospital pharmacist | Refractory symptom treatment (WG 2) – WG member |
| Cindy Joosen | Nurse practitioner specialized in paediatric palliative care | Refractory symptom treatment (WG 2) – WG member  Dyspnoea (WG 1C) – Reviewer  Death Rattle (WG 1J) - Reviewer |
| Linda Corel | Paediatrician, intensivist | Refractory symptom treatment (WG 2) – WG member  Dyspnoea (WG 1C) – Reviewer  Haematological symptoms (WG 1D) – Reviewer  Coughing (WG 1E) - Reviewer  Death Rattle (WG 1J) - Reviewer |
| Ilse Zaal-Schuller | Physician intellectual disabilities | Refractory symptom treatment (WG 2) – WG member  ACP and SDM (WG 3) – Reviewer |
| Hilda Mekelenkamp | Paediatric nurse | Refractory symptom treatment (WG 2) – WG member |
| Suzanne van de Vathorst | Professor medical ethics | Refractory symptom treatment (WG 2) – WG member |
| Loes Berkhout | Spiritual caregiver | ACP and SDM (WG 3) – WG member  Psychological interventions (WG 5A) - Reviewer  Spiritual support (WG 5C) - Reviewer |
| Rosa Geurtzen | Neonatologist | ACP and SDM (WG 3) – WG member |
| Nellie van Wageningen | Medical pedagogical care provider | Psychological interventions (WG 5A) – WG member  Social and Practical Support (WG 5B) – WG member |
| Carolien Huizinga | Policy advisor | Organisation of care (WG 4) – WG member |
| Mara van Stiphout | Policy officer knowledge and education | Organisation of care (WG 4) – WG member |
| Liesbeth Rietveld | Nurse Child Comfort Team | Organisation of care (WG 4) – WG member |
| Tanja van Roosmalen | Grief counsellor, orthopedagogue | Loss and Bereavement (WG 6) – WG member |
| **WG reviewers** | | |
| Bas Oude Ophuis | Child and youth psychiatrist | Anxiety and Depression (WG 1A) – Reviewer |
| Lisette ‘t Hart-Kerkhoffs | Child and youth psychiatrist | Delirium (WG 1B) – Reviewer |
| Carla Juffermans | General practitioner, physician palliative care | Dyspnoea (WG 1C) – Reviewer  Haematological symptoms (WG 1D) – Reviewer  Coughing (WG 1E) - Reviewer |
| Emmy Räkers | General practitioner, physician intellectual disabilities | Neurological symptoms (WG 1H) – Reviewer |
| Henriette Sjouwke | Physician palliative care | Neurological symptoms (WG 1H) – Reviewer |
| Tom de Leeuw | Paediatric anaesthesiologist | Pain (WG 1I) - Reviewer |
| Jennifer van Dijk | Psychologist | Pain (WG 1I) - Reviewer |
| Anne Weenink | Medical pedagogical care provider | Pain (WG 1I) – Reviewer  Refractory symptom treatment (WG 2) – Reviewer  ACP and SDM (WG 3) – Reviewer  Organisation of care (WG 4) – Reviewer |
| Elise van de Putte | Paediatrician - social paediatrics | Pain (WG 1I) – Reviewer  Fatigue (WG 1K) - Reviewer |
| Hanneke Heinen | Medical pedagogical care provider | Fatigue (WG 1K) - Reviewer |
| Anne Haag | Medical pedagogical care provider | Psychological interventions (WG 5A) – Reviewer  Social and Practical Support (WG 5B) – Reviewer |
| Annelies Gijsbertsen-Kool | Paediatric nurse | Loss and Bereavement (WG 6) – Reviewer |

**Patient representative panel**

| Name | Role |
| --- | --- |
| Marguerite Gorter-Stam | Parent |
| Suzan Mulder | Parent |
| Elisabeth Bruinja | Parent |
| Mark Mooij | Parent |
| José Bakker | Parent |
| Irma van Leeuwen | Parent |
| Jelke van Hoorn | Parent |
| Petra den Hollander | Parent |
| Marie-José Pulles | Parent |

# Appendix D. Working structure for guideline development


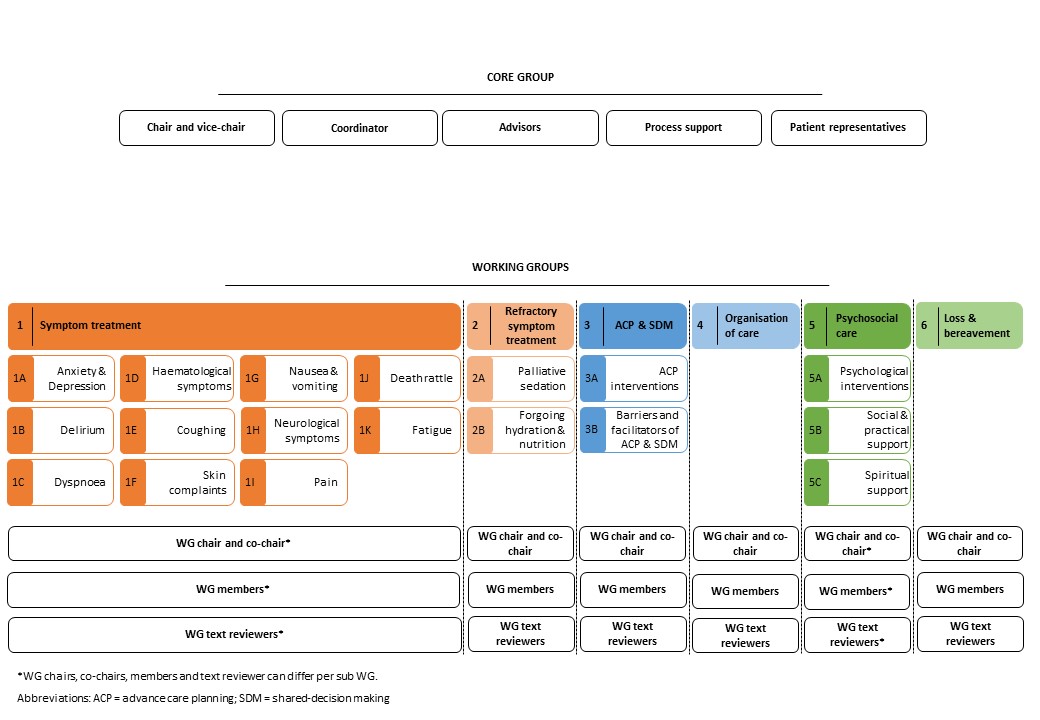


# Appendix E. Guideline development process


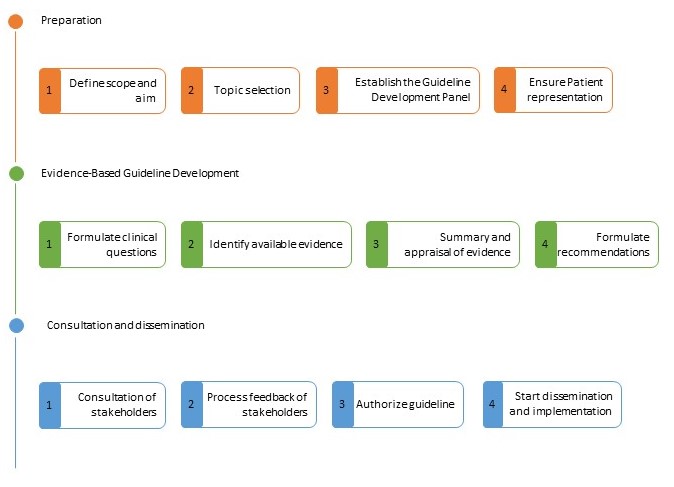


# Appendix F. Clinical questions

**Identification of evidence – Quantitative studies**

WG 1 Symptom treatment

Sub-WG 1A Anxiety and Depression

1. What is most effective non pharmacological intervention for anxiety and depression in children aged 0 to 18 years in the palliative phase?
2. What is most effective pharmacological intervention for anxiety and depression in children aged 0 to 18 years in the palliative phase?

Sub-WG 1B Delirium

1. What is most effective non pharmacological intervention for delirium in children aged 0 to 18 years in the palliative phase?
2. What is most effective pharmacological intervention for delirium in children aged 0 to 18 years in the palliative phase?

Sub-WG 1C Dyspnoea

1. What is most effective non pharmacological intervention for dyspnoea in children aged 0 to 18 years in the palliative phase?
2. What is most effective pharmacological intervention for dyspnoea in children aged 0 to 18 years in the palliative phase?

Sub-WG 1D Haematological symptoms

1. What the most effective pharmacological interventions for anaemia in children aged 0 to 18 in the palliative phase?
2. What is the most effective pharmacological intervention for thrombocytopenia in children aged 0 to 18 in the palliative phase?
3. What is the most effective pharmacological intervention for haemorrhages in children aged 0 to 18 in the palliative phase?
4. What is the most effective pharmacological intervention for thrombosis in children aged 0 to 18 in the palliative phase?

Sub-WG 1E Coughing

1. What is most effective non pharmacological intervention for coughing in children aged 0 to 18 years in the palliative phase?
2. What is most effective pharmacological intervention for coughing in children aged 0 to 18 years in the palliative phase?

Sub-WG 1F Skin Complaints

1. What is most effective non pharmacological intervention for skin complaints (itching, pressure ulcers and wounds) in children aged 0 to 18 years in the palliative phase?
2. What is most effective pharmacological intervention for skin complaints (itching, pressure ulcers and wounds) in children aged 0 to 18 years in the palliative phase?

Sub-WG 1G Nausea and Vomiting

1. What is most effective non pharmacological intervention for nausea and vomiting in children aged 0 to 18 years in the palliative phase?
2. What is most effective pharmacological intervention for nausea and vomiting in children aged 0 to 18 years in the palliative phase?

Sub-WG 1H Neurological symptoms

1. What is most effective non pharmacological intervention for neurological symptoms (epilepsy, spasticity, dyskinesia syndromes, loss of neurological function) in children aged 0 to 18 years in the palliative phase?
2. What is most effective pharmacological intervention for neurological symptoms (epilepsy, spasticity, dyskinesia syndromes, loss of neurological function) in children aged 0 to 18 years in the palliative phase?

Sub-WG 1I Pain

1. What is most effective non pharmacological intervention for pain in children aged 0 to 18 years in the palliative phase?
2. What is most effective pharmacological intervention for pain in children aged 0 to 18 years in the palliative phase?

Sub-WG 1J Death rattle

1. What is most effective non pharmacological intervention for death rattle in children aged 0 to 18 years in the palliative phase?
2. What is most effective pharmacological intervention for death rattle in children aged 0 to 18 years in the palliative phase?

Sub-WG 1K Fatigue

1. What is most effective non pharmacological intervention for fatigue in children aged 0 to 18 years in the palliative phase?
2. What is most effective pharmacological intervention for fatigue in children aged 0 to 18 years in the palliative phase?

WG2. Refractory symptoms

Sub-WG 2A Palliative sedation

1. What is the effect of palliative sedations with other medication than midazolam (possibly in combination with morphine) in children aged 0 to 18 years in the terminal phase on quality of life and lifespan?
2. What is the effect of palliative sedations with other medication than midazolam (possibly in combination with morphine) in children with multiple severe disabilities aged 0 to 18 years in the terminal phase on quality of life and lifespan?

Sub-WG 2B Forgoing hydration & nutrition

1. What is the effect of hydration and nutrition deprivation in children aged 0 to 18 years in the terminal phase on quality of life, life span and quality of life of parents?

WG 3 Advance care planning and shared decision-making

Sub-WG 3A Advance care planning interventions

1. What is the effect of Advance Care Planning for children aged 0 to 18 in the palliative phase and their parents and family members on decision making and quality of life.

WG 4 Organisation of care

1. What is the effect of interventions for improving continuity and coordination of care for children aged 0 to 18 in the palliative phase?

WG 5 Psychosocial care

Sub-WG 5A Psychological interventions

1. What is the effect of psychological intervention for children aged 0 to 18 years in the palliative phase?
2. What is the effect of psychological intervention for parents and family members of children aged 0 to 18 years in the palliative phase?

Sub-WG 5B Social and practical support

1. What forms of social and practical support are considered as effective by children aged 0 to 18 years in the palliative phase and their parents and family members?

Sub-WG 5C Cultural, Spiritual and Religious support

1. What forms of cultural, spiritual and religious support are considered as effective by children aged 0 to 18 years in the palliative phase and their parents and family members?

WG 6 Loss and Bereavement

1. What is the effect of bereavement care interventions for children aged 0 to 8 years in the palliative phase and their parents and family members and caregivers?
2. Which components are used in bereavement care interventions?
3. What are the experiences and needs of parents and caregivers with regards to components of bereavement care interventions?
4. Which communicative and affective strategies can support parents during the end of life and after the death of their child?

**Identification of evidence – qualitative studies**

WG 3 Advance care planning and shared decision-making

Sub-WG 3B Barriers and facilitators of advance care planning and shared decision-making

1. What are barriers and facilitators to children aged 0 to 18 years in the palliative phase and their parents, family members and health care professionals in being involved in Advance Care Planning and Shared Decision Making.

# Appendix G. Search strategies

**Identification of quantitative evidence**

Search strategy

*Search A*

Search date October 5, 2018.

Databases OVID Medline, OVID PreMedline, Cochrane Library (CDSR & CENTRAL)

Search limits Publication date: 2010-present;

Language: English and Dutch;

Study design: Randomized Controlled Trials (RCTs), Clinical Controlled Trials (CCTs), and Systematic Reviews (of RCTs and/or CCTs).

*Search B*

Search date 24-01-2020

Databases PubMed (Medline)

Search limits Publication date: 2018 – present;

Language: English and Dutch;

Study design: Randomized Controlled Trials (RCTs), Clinical Controlled Trials (CCTs), and Systematic Reviews (of RCTs and/or CCTs).

Search strings

*Search A*

| Name researcher: | Joan Vlaayen |
| --- | --- |
| Search date: | 05-10-2018 |
| **Medline (OVID)** | |
| Search 1:  Intervention | 1. exp Palliative Care/ (49362) or 2. palliat*.tw. (58051) or 3. advanced disease*.tw. (15975) or 4. (end-stage disease* or end stage disease* or end-stage illness or end stage).tw. (52291) or 5. Terminally Ill/ (6207) or 6. Terminal Care/ (25841) or 7. (terminal* adj6 care*).tw. (3554) or 8. ((terminal* adj6 ill*) or terminal-stage* or dying or (close adj6 death)).tw. (37618) or 9. (terminal* adj6 disease*).tw. (3457) or 10. (end adj6 life).tw. (19644) or 11. hospice*.tw. (9908) |
| Search 2:  Population | 1. exp Infant/ (1075702) or 2. exp Child/ (1789770) or 3. Adolescent/ (1887787) or 4. (minors* or perinat* or postnat* or kid or kids or neonat* or newborn* or infan* or child* or adoles* or paediatric* or pediatric* or baby* or babies or toddler* or teen* or juvenil* or boy* or girl* or underag* or youth* or kindergar* or puber* or pubescen* or schools or nursery school* or preschool* or primary school* or secondary school* or elementary school* or high school* or highschool* or school age* or schoolage*).mp. (3978287) |
| Search 3:  Study design | 1. randomized controlled trial.pt. (468961) or 2. controlled clinical trial.pt. (92654) or 3. randomi?ed.ab. (442505) or 4. placebo.ab. (175141) or 5. clinical trials as topic.sh. (184906) or 6. randomly.ab. (255868) or 7. trial.ti. (163102) NOT 8. (exp animals/ not humans.sh. (4501441)) |
| Search 4:  Study design | 1. meta-analysis.mp,pt. or review.pt. or search:.tw. (2504080) |
| Combined: | Search 1 AND Search 2 AND (SEARCH 3 OR SEARCH 4) |
| Total records: | 2045 records |
| **PreMedline (OVID)** | |
| Search 1:  Intervention | 1. palliat*.tw. (7228) or 2. advanced disease*.tw. (1855) or 3. (end-stage disease* or end stage disease* or end-stage illness or end stage).tw. (6487) or 4. (terminal* adj6 care*).tw. (243) or 5. ((terminal* adj6 ill*) or terminal-stage* or dying or (close adj6 death)).tw. (3125) or 6. (terminal* adj6 disease*).tw. (237) or 7. (end adj6 life).tw. (2679) or 8. hospice*.tw. (944) |
| Search 2:  Population | 1. (minors* or perinat* or postnat* or kid or kids or neonat* or newborn* or infan* or child* or adoles* or paediatric* or pediatric* or baby* or babies or toddler* or teen* or juvenil* or boy* or girl* or underag* or youth* or kindergar* or puber* or pubescen* or schools or nursery school* or preschool* or primary school* or secondary school* or elementary school* or high school* or highschool* or school age* or schoolage*).mp. (195151) |
| Search 3:  Study design | 1. randomized controlled trial.pt. (277) or 2. controlled clinical trial.pt. (20) or 3. randomi?ed.ab. (51918) or 4. placebo.ab. (13994) or 5. randomly.ab. (36549) or 6. trial.ti. (20815) |
| Search 4:  Study design | 1. meta-analysis.mp,pt. or review.pt. or search:.tw. (170831) |
| Combined: | Search 1 AND Search 2 AND (SEARCH 3 OR SEARCH 4) |
| Total records: | 301 records |
| **Cochrane library (Cochrane Database of Systematic Reviews and CENTRAL)** | |
| Search 1:  Intervention | 1. MeSH descriptor: [Palliative Care] explode all trees (1454) OR 2. palliat*:ti,ab (4487) OR 3. advanced disease*:ti,ab (15208) OR 4. (end-stage disease* or end stage disease* or end-stage illness or end stage):ti,ab (8120) OR 5. MeSH descriptor: [Terminally Ill] explode all trees (80) OR 6. MeSH descriptor: [Terminal Care] explode all trees (407) OR 7. (terminal* NEAR/6 care*):ti,ab (110) OR 8. ((terminal* NEAR/6 ill*) or terminal-stage* or dying or (close NEAR/6 death)):ti,ab (1290) OR 9. (terminal* NEAR/6 disease*):ti,ab (143) OR 10. (end NEAR/6 life) (1966) OR 11. hospice*:ti,ab (529) |
| Search 2:  Population | 1. MeSH descriptor: [Infant] explode all trees (15033) OR 2. MeSH descriptor: [Child] explode all trees (1416) OR 3. MeSH descriptor: [Adolescent] explode all trees (97673) OR 4. (minors* or perinat* or postnat* or kid or kids or neonat* or newborn* or infan* or child* or adoles* or paediatric* or pediatric* or baby* or babies or toddler* or teen* or juvenil* or boy* or girl* or underag* or youth* or kindergar* or puber* or pubescen* or schools or nursery school* or preschool* or primary school* or secondary school* or elementary school* or high school* or highschool* or school age* or schoolage*):ti,ab (150225) |
| Combined: | Search 1 AND Search 2 |
| Total records: | Cochrane Database of Systematic reviews: 246 reviews; CENTRAL: 1496 |

*Search B*

| Name researcher | Kim van Teunenbroek |
| --- | --- |
| Search date: | 24-01-2020 |
| **PubMed (Medline)** | |
| Search 1:  Intervention | palliative care OR Care, Palliative OR Palliative Treatment OR Palliative Treatments OR Treatment, Palliative OR Therapy, Palliative OR Palliative Therapy OR Palliative Medicine OR Medicine, Palliative OR OR pediatric palliative care OR children's hospice OR Hospice care OR Terminal care OR “end life” |
| Search 2:  Population | infant OR infan* OR newborn OR newborn* OR new-born* OR baby OR baby* OR babies OR neonat* OR perinat* OR postnat* OR child OR child* OR schoolchild* OR schoolchild OR school child OR school child* OR kid OR kids OR toddler* OR adolescent OR adoles* OR teen* OR boy* OR girl* OR minors OR minors* OR underag* OR under ag* OR juvenil* OR youth* OR kindergar* OR puberty OR puber* OR pubescen* OR prepubescen* OR prepuberty* OR pediatrics OR pediatric* OR paediatric* OR peadiatric* OR schools OR nursery school* OR preschool* OR pre school* OR primary school* OR secondary school* OR elementary school* OR elementary school OR high school* OR highschool* OR school age OR schoolage OR school age* OR schoolage* OR infancy OR schools, nursery OR infant, newborn |
| Search 3: study design | (systematic review[tiab] OR review literature[mh] OR review[tiab] OR reviews[tiab] OR review[pt] OR systematic literature review[tiab] OR systematic literature review[tiab]) OR (meta analysis[pt] OR meta analysis[mh] OR meta analysis[tiab] OR metaanalysis[tiab] OR meta analyses[tiab]) OR (guidelin*[tiab]) |
| Search 4:  Study design | (randomized controlled trial [pt] OR controlled clinical trial [pt] OR randomized [tiab] OR placebo [tiab] OR drug therapy [sh] OR randomly [tiab] OR trial [tiab] OR groups [tiab]) NOT (animals [mh] NOT humans [mh]) |
| Combined: | Search 1 AND Search 2 AND (Search 3 OR Search 4) |
| Total records: | 990 |

**Identification of qualitative evidence**

Search strategy

*Included literature from the NICE-guideline*

We used the literature as cited in the NICE-guideline ‘End of life care for infants, children and young people with life-limiting conditions: planning and management’. The original search strategy, inclusion criteria and excluded studies can be found in [Appendix A – F and H – J](https://www.nice.org.uk/guidance/ng61/evidence/appendices-af-and-hj-pdf-2728081262).

*Search*

Search date 16-09-2020

Databases PubMed (Medline)

Search limits Publication date: 2016 - present;

Language: English;

Study design: Qualitative studies, Mixed-methods observational studies where qualitative data were reported, Systematic reviews of qualitative and mixed-methods observational studies.

Search strings

*Search A*

Original search strings performed by the National Institute of Health Care excellence are reported in [Appendix E3: Search strategies, Advance Care Plan](https://www.nice.org.uk/guidance/ng61/evidence/appendices-af-and-hj-pdf-2728081262) (p.127-130) of the NICE-guideline ‘End of life care for infants, children and young people with life-limiting conditions: planning and management’.

*Search B*

| Name Researcher | Kim van Teunenbroek and Dayna van Heel |
| --- | --- |
| Search date | 16-9-2020 |
| **PubMed (MEDLINE)** | |
| Search 1: Patient | infant OR infan* OR newborn OR newborn* OR new-born* OR baby OR baby* OR babies OR neonat* OR perinat* OR postnat* OR child OR child* OR schoolchild* OR schoolchild OR school child OR school child* OR kid OR kids OR toddler* OR adolescent OR adoles* OR teen* OR boy OR boys OR girl* OR minors OR minors* OR underag* OR under age OR juvenil* OR youth* OR kindergar* OR puberty OR puber* OR pubescen* OR prepubescen* OR prepuberty* OR pediatrics OR pediatric* OR paediatric* OR peadiatric* OR schools OR nursery school* OR preschool* OR pre school* OR primary school* OR secondary school* OR elementary school* OR elementary school OR high school* OR highschool* OR school age OR schoolage OR schoolage* OR infancy OR schools, nursery OR infant, newborn |
| Search 2: Patient | palliative care OR Care, Palliative OR Palliative Treatment OR Palliative Treatments OR Treatment, Palliative OR Therapy, Palliative OR Palliative Therapy OR Palliative Medicine OR Medicine, Palliative OR pediatric palliative care OR children's hospice OR Hospice care OR Terminal care OR “end life” |
| Search 3: Intervention | "patient care planning"[MeSH Terms] OR Patient care planning[tiab] OR "advance care planning"[MeSH Terms] OR advance* care plan*[tiab] OR "Advance Directives"[Mesh] OR advance directive*[tiab] OR "living wills"[MeSH Terms] OR living will*[tiab] OR "Case Management"[Mesh] OR case management [tiab] OR "critical pathways"[MeSH Terms] OR critical pathways*[tiab] OR critical path*[tiab] OR parallel plan*[tiab] OR "resuscitation orders"[MeSH Terms] OR resuscitation order*[tiab] OR resuscitat* order*[tiab] OR resuscitat* policy[tiab] OR resuscitat* policies[tiab] OR resuscitat* decision[tiab] OR resuscitat attempt*[tiab] OR "withholding treatment"[MeSH Terms] OR withhold* treatment*[tiab] OR withdraw* treatment [tiab] OR "euthanasia, passive"[MeSH Terms] OR passive euthanasia[tiab] OR "Decision Making, Shared"[Mesh] OR "Clinical Decision-Making"[Mesh] OR shared decision making[tiab] OR shared decision-making[tiab] OR clinical decision-making[tiab] OR clinical decision making[tiab] OR medical decision making[tiab] OR medical decision-making[tiab] OR decision*[tiab] OR "Decision Making"[Mesh] OR decision making[tiab] OR "Advance Directive Adherence"[Mesh] OR directive* Adhere*[tiab] OR directive* compl*[tiab] |
| Search 4: Study design | "Letter"[Publication Type] OR "Editorial"[Publication Type] OR "News"[Publication Type] OR "Historical Article"[Publication Type] OR "Anecdotes as Topic"[Mesh] OR "Comment" [Publication Type] OR "Qualitative Research"[Mesh] OR "Interviews as Topic"[Mesh] OR "Focus Groups"[Mesh] OR "Case Reports"[Publication Type] OR qualitative stud*[tiab] OR qualitative[tiab] OR qualitative research[tiab] OR interview*[tiab] OR Case Report*[tiab] OR focus group*[tiab] |
| Search 5: Study design | "Randomized Controlled Trial" [Publication Type] OR random*[tiab] |
| Combined: | Search 1 AND 2 AND 3 and (4 NOT 5) |
| Total eligible records | 1232 |

**Identification of additional literature: guidelines**

Search strategy

Search date 24-01-2020

Databases Guideline International Network (GIN), IPOG, NICE, NVK en Pallialine.

Search limits Publication date: 2010-present;

Language: English and Dutch only.

Search strings

| Name researcher: | Kim van Teunenbroek |
| --- | --- |
| Search date: | 24-01-2020 |
| **Systematic search in GIN** | |
| Search 1:  Intervention | palliative care OR pediatric palliative care OR children's hospice |
| Search 2:  Population | child OR child* OR children* |
| Combined: | Search 1 AND Search 2 |
| Total records | 362 |
| **Focused search in databases of NICE, IPOG, NVK and Pallialine** | |
| Search NICE database | Guidelines on care for children, n = 1 |
| Search IPOG database | Guidelines on palliative care for children, n = 2 |
| Search NVK database | Guidelines on care for children, n = 2 |
| Search Pallialine database | Guidelines on palliative care for adults, n = 11 |
| Referencing | Guidelines on palliative care for children, n = 1  Guidelines on care for children, n = 6  Guidelines on palliative care for adults, n = 4 |
| Total records: | 27 |

# Appendix H. Inclusion criteria

**Identification of quantitative evidence**

| Patient  population | - Children with a life threatening or life limiting conditions according to the definition of the World Health Organisation (WHO)* aged 0 to 18 years old. This includes children with:   - acute life-threatening conditions from which recovery may or may not be possible,   - chronic life-threatening conditions that may be cured or controlled for a long period but also may cause death,   - progressive life-threatening conditions for which no curative treatment is available,   - severe neurologic conditions that are not progressive but may cause deterioration and death. - Patient population consisting of more than 10 patients. - 75% of the patient population consists of children aged 0 to 18 years old. |
| --- | --- |
| Intervention | All palliative interventions on:   - Treatment of anxiety and depression, delirium, dyspnoea, haematological symptoms, coughing, skin complaints, nausea and vomiting, pain, neurological symptoms and fatigue - Treatment of refractory symptoms (palliative sedation and forgoing hydration & nutrition) - Advance Care Planning and shared decision making. - Organisation of care. - Psychosocial care: psychological interventions, social and practical support, cultural, spiritual and religious support. - Loss and bereavement |
| Study design | - Randomized Controlled Trials (RCTs) or Controlled Clinical Trials (CCTs). - Systematic reviews (of RCTs and/or CCTs). |
| Study characteristics | - English or Dutch language. - Minimum of two authors. - Abstract is available. |
| *World Health Organization. Integrating palliative care and symptom relief into paediatrics: a WHO guide for health-care planners, implementers and managers. 2018 | |

**Identification of qualitative evidence**

| Patient population | - Children with a life threatening or life limiting conditions according to the definition of the World Health Organisation (WHO)* aged 0 to 18 years old. This includes children with:   - acute life-threatening conditions from which recovery may or may not be possible,   - chronic life-threatening conditions that may be cured or controlled for a long period but also may cause death,   - progressive life-threatening conditions for which no curative treatment is available,   - severe neurologic conditions that are not progressive but may cause deterioration and death. - Parents, carers and family members (brothers and sisters) of children with a life threatening or life limiting disease aged 0 to 18 years old. - Health care professionals of children with a life threatening or life limiting disease aged 0 to 18 years old. |
| --- | --- |
| Subject | - Advance care planning. - Shared decision-making. |
| Outcome measure | - Barriers and facilitators. |
| Study design | - Qualitative studies (for example ethnographic studies, interviews, focus groups) - Mixed-methods observational studies where qualitative data were reported (for example survey studies) - Systematic reviews of qualitative and mixed-methods observational studies. |
| Study characteristics | - English or Dutch. - Minimum of two authors. - Abstract is available. |
| *World Health Organization. Integrating palliative care and symptom relief into paediatrics: a WHO guide for health-care planners, implementers and managers. 2018 | |

**Identification of additional literature: guidelines**

| Patient population and study design | - Children with a life threatening or life limiting conditions according to the definition of the World Health Organisation (WHO)* aged 0 to 18 years old. This includes children with:   - acute life-threatening conditions from which recovery may or may not be possible,   - chronic life-threatening conditions that may be cured or controlled for a long period but also may cause death,   - progressive life-threatening conditions for which no curative treatment is available,   - severe neurologic conditions that are not progressive but may cause deterioration and death. - Guidelines on palliative care for adults with separate recommendations on palliative care for children. |
| --- | --- |
| Subjects | Guidelines on:   - Treatment of anxiety and depression, delirium, dyspnoea, haematological symptoms, coughing, skin complaints, nausea and vomiting, pain, neurological symptoms and fatigue - Treatment of refractory symptoms (palliative sedation and forgoing hydration & nutrition) - Advance Care Planning and shared decision making. - Organisation of care. - Psychosocial care: psychological interventions, social and practical support, cultural, spiritual and religious support. - Loss and bereavement |
| Exceptions | - When paediatric palliative care guidelines on (refractory)symptom treatment were not available or inadequate, guidelines on symptom treatment in general paediatrics or adult palliative care* were included. - When paediatric palliative care guidelines on advance care planning, shared decision-making, organisation of care, psychosocial care and loss and bereavement were not available or inadequate, guidelines on palliative care for adults** were included. |
| *World Health Organization. Integrating palliative care and symptom relief into paediatrics: a WHO guide for health-care planners, implementers and managers. 2018)  **Guidelines are only included if the recommendations were considered relevant for children in the palliative phase | |

# Appendix I. Criteria for appraisal of evidence and strength of recommendations.

| **Grade of Recommendation**  **Conclusions of evidence according to GRADE** | **Strong recommendation**  **to do**  Benefits >>> risk & harms | **Moderate**  **recommendation**  **to do**  Benefits > or = risk & harms | **Recommendation**  **not to do**  No benefit / Potentially harm |
| --- | --- | --- | --- |
| **High quality of evidence**  Consistent evidence from well performed and high quality studies or systematic reviews (low risk of bias, direct, consistent, precise). | Strong recommendation based on high quality evidence | Moderate recommendation based on high quality evidence | Recommendation not to do based on high quality evidence |
| **Moderate quality of evidence**  Evidence from studies or systematic reviews with few important limitations. | Strong recommendation based on moderate quality evidence | Moderate recommendation based on moderate quality evidence | Recommendation not to do based on moderate quality evidence |
| **Low to very low quality of evidence**  Evidence from studies with serious flaws, only expert opinion, or standards of care. | Strong recommendation based on expert opinion | Moderate recommendation based on (very) low quality evidence  Diverging expert opinions | Recommendation not to do based on expert opinion |
|  | **Wording in recommendations:** | |  |
|  | We strongly recommend … | We moderately recommend … | We do not recommend … |

Gibbons RJ, Smith S, Antman E. American College of Cardiology/American Heart Association clinical practice guidelines: Part I: where do they come from? Circulation. 2003; 107(23): 2979-86.

# Appendix J. Results: identified evidence and selected additional literature

**Identification of quantitative evidence**

| **Year** | **Bibliography** | **Study design** |
| --- | --- | --- |
| 2015 | ***Beecham E et al.*** Pharmacological interventions for pain in children and adolescents with life-limiting conditions. Cochrane Database of Systematic Reviews 2015 3(13) | Systematic review of RCTs |
| 2015 | ***Eccleston C et al.*** Psychological interventions for parents of children and adolescents with chronic illness. Cochrane Database of Systematic Reviews 2015 4)^2^ | Systematic review of RCTs |
| 2014 | ***Goldbeck L et al.*** Psychological interventions for individuals with cystic fibrosis and their families. Cochrane Database of Systematic Reviews 2014 6) | Systematic review of RCTs |
| 2011 | ***Wiffen PJ et al.*** Opioids for cancer‐related pain in children and adolescents. Cochrane Database of Systematic Reviews 2017 7): The Journal of Clinical Endocrinology and Metabolism 2011;96(2):355–64. | Systematic review of RCTs |
| 2019 | ***Rosenberg AR et al.*** Hope and benefit finding: Results from the PRISM randomized controlled trial. Pediatr Blood Cancer 2019 66 (1): e27485 | RCT |
| 2019 | ***Rosenberg AR et al.*** Effect of the Promoting Resilience in Stress Management Intervention for Parents of Children With Cancer (PRISM-P): A Randomized Clinical Trial. JAMA Netw Open 2019 2 (9): e1911578 | RCT |
| 2019 | ***Steineck A et al.*** A Psychosocial Intervention's Impact on Quality of Life in AYAs with Cancer: A Post Hoc Analysis from the Promoting Resilience in Stress Management (PRISM) Randomized Controlled Trial. Children (Basel) 2019 6 (11) | RCT |
| 2017 | ***Lyon ME et al.*** A randomized clinical trial of adolescents with HIV/AIDS: pediatric advance care planning. AIDS Care. 2017;29(10):1287-96. | RCT |
| 2016 | ***Beheshtipour N et al.*** The Effect of Educational-spiritual Intervention on The Burnout of The Parents of School Age Children With Cancer: A Randomized Controlled Clinical Trial. IJCBNM January 2016; Vol 4,No 1^1^ | RCT |
| 2016 | ***Borjalilu S et al.*** Spiritual care Training for Mothers of Children with Cancer: Effects on Quality of Care and Mental Health of Caregivers. Asian Pac J Cancer Prev, 17 (2), 545-552, 2016 ^1^ | RCT |
| 2015 | ***Raitio K et al.*** Evaluating a bereavement follow-up intervention for grieving mothers after the death of a child. Scand J Caring Sci. 2015 Sep;29(3):510-20 ^1^ | RCT |
| 2014 | ***Copeland I et al.*** Botulinum toxin A for nonambulatory children with cerebral palsy: a double blind randomized controlled trial. J Pediatr 2014;165:140-6). | RCT |
| 2014 | ***Lima C et al.*** Effects of noninvasive ventilation on treadmill 6-min walk distance and regional chest wall volumes in cystic fibrosis: Randomized controlled trial. Respir Med 2014; 108:1460–1468^2^ | RCT |
| 2014 | ***Lyon ME et al.*** A longitudinal, randomized, controlled trial of advance care planning for teens with cancer: anxiety, depression, quality of life, advance directives, spirituality. J Adolesc Health. 2014;54(6):710-7 | RCT |
| 2013 | ***Lyon ME et al.*** Family-centered advance care planning for teens with cancer. Jama, Pediatr. 2013;167(5):460-7. | RCT |
| 2010 | ***Lyon ME et al.*** Is it safe? Talking to teens with HIV/AIDS about death and dying: a 3-month evaluation of Family Centered Advance Care (FACE) planning - anxiety, depression, quality of life. HIV/AIDS Research and Palliative Care. 2010;2:27-37. | RCT |
| 2010 | ***Olesch CA et al.*** Repeat botulinum toxin-A injections in the upper limb of children with hemiplegia: a randomized controlled trial, Developmental Medicine and Child Neurology, 52, 79-86, 2010 ^2^ | RCT |
| 2009 | ***Gore L et al.*** Aprepitant in adolescent patients for prevention of chemotherapy-induced nausea and vomiting: a randomized, double-blind, placebo-controlled study of efficacy and tolerability. Pediatr Blood Cancer 2009;52:242–247 | RCT |
| 2007 | ***Riad, W. et al.*** Effect of midazolam, dexamethasone and their combination on the prevention of nausea and vomiting following strabismus repair in children. European Journal of Anaesthesiology 2007; 24: 697-701 | RCT |
| 2006 | ***Razouk BI et al.*** Double-Blind, Placebo-Controlled Study of Quality of Life, Hematologic End Points, and Safety of Weekly Epoetin Alfa in Children With Cancer Receiving Myelosuppressive Chemotherapy. J Clin Oncol 2006; 24:3583-3589. | RCT |
| 2005 | ***Maxwell LG et al.*** The effects of a Small-Dose Naloxone Infusion on Opioid-Induced Side Effects and Analgesia in Children and Adolescents Treated with Intravenous Patient-Controlled Analgesia: A Double-Blind, Prospective, Randomized, Controlled Study. Anesth Analg 2005;100:953–8 | RCT |
| 2002 | ***Buyukpamukcu M et al.*** Is Epoetin Alfa a treatment option for chemotherapy-related anaemia in children? Med Pediatr Oncol 2002;29 (4):455-8 | RCT |
| 2001 | ***Aksoylar S et al.*** Comparison of tropisetron and granisetron in the control of nausea and vomiting in children receiving combined cancer chemotherapy. Pediatr Hematol Oncol 2001 Sep;18(6):397-406. | RCT |
| 2001 | ***De jong W et al.*** Inspiratory muscle training in patients with cystic fibrosis. RESPIRATORY MEDICINE (2001) 95, 31–36^2^ | RCT |
| 1999 | ***Parker RI et al***. Randomized, double-blind, crossover, placebo-controlled trial of intravenous ondansetron for the prevention of intrathecal chemotherapy-induced vomiting in children. Biol Blood Marrow Transplant 1999;5(6):386-93 | RCT |
| 1998 | ***Kóseoglu V et al.*** Comparison of the efficacy and side-effects of ondansetron and metoclopramide-diphenhydramine administered to control nausea and vomiting in children treated with antineoplastic chemotherapy: a prospective randomized study. Eur J Pediatr 1998 Oct;157(10):806-10 | RCT |
| 1996 | ***Brock P et al. An*** increased loading dose of ondansetron: a north european, double-blind randomised study in children, comparing 5 mg/m2 with 10 mg/m2. Eur J Cancer 1996 Sep;32A(10):1744-8 | RCT |
| 1994 | ***Jacknow DS et al.*** Hypnosis in the prevention of chemotherapy-related nausea and vomiting in children: a prospective study. J Dev Behav Pediatr 1994;15(4):258-64 | RCT |
| 1994 | ***Orchard PJ et al.*** A prospective randomized trial of the anti-emetic efficacy of ondansetron and granisetron during bone marrow transplantation. J Dev Behav Pediatr 1994;15(4):258-64 | RCT |

**Identification of qualitative evidence**

| **Year** | **Bibliography** | **Study design** |
| --- | --- | --- |
| 2021 | ***Fahner et al.*** Evaluation showed that stakeholders valued the support provided by the Implementing Pediatric Advance Care Planning Toolkit. Acta Paediatr 2021;110:237-46. | Qualitative study |
| 2020 | ***Edwards et al.*** Decisions for long-term ventilation for children: perspectives of family members. Ann Am Thorac Soc 2020;17:72-80. | Qualitative study |
| 2020 | ***Fahner et al.*** Towards advance care planning in pediatrics: a qualitative study on envisioning the future as parents of a seriously ill child. Eur J Pediatr 2020;17:1461-68. | Qualitative study |
| 2020 | ***Hein et al.*** Identifying key elements for paediatric advance care planning with parents, healthcare providers and stakeholders: A qualitative study. Palliat Med 2020;34:300-8. | Qualitative study |
| 2020 | ***Lord et al.*** Assessment of Bereaved Caregiver Experiences of Advance Care Planning for Children With Medical Complexity. JAMA Netw Open 2020;3:e2010337. | Qualitative study |
| 2020 | ***Mekelenkamp et al.*** Parental experiences in end-of-life decision-making in allogeneic pediatric stem cell transplantation: "Have I been a good parent?". Pediatr Blood Cancer 2020;67:e28229. | Qualitative study |
| 2020 | ***Orkin et al.*** Toward an Understanding of Advance Care Planning in Children With Medical Complexity. Pediatrics 2020;145:e20192241. | Qualitative study |
| 2020 | ***Sisk et al.*** Communication in Pediatric Oncology: A Qualitative Study. Pediatrics 2020;146:e20201193. | Qualitative study |
| 2019 | ***Sasazuki et al.*** Decision-making dilemmas of paediatricians: a qualitative study in Japan. BMJ Open 2019;9:e026579. | Qualitative study |
| 2019 | ***Mitchell et al.*** Parental experiences of end of life care decision-making for children with life-limiting conditions in the paediatric intensive care unit: a qualitative interview study. BMJ Open 2019;9:e028548. | Qualitative study |
| 2018 | ***Day et al.*** "We just follow the patients' lead": Healthcare professional perspectives on the involvement of teenagers with cancer in decision making. Paediatric Blood Cancer 2018;65. | Qualitative study |
| 2018 | ***Jack et al.*** A qualitative study of health care professionals' views and experiences of paediatric advance care planning. BMC Palliat Care 2018;17:93. | Qualitative study |
| 2018 | ***Murrell et al.*** Identifying Opportunities to Provide Family-centered Care for Families With Children With Type 1 Spinal Muscular Atrophy. J Pediatr Nurs 2018;43:111-9. | Qualitative study |
| 2018 | ***Superdock et al.*** Exploring the vagueness of Religion & Spirituality in complex paediatric decision-making: a qualitative study. BMC Palliat Care 2018;17:107. | Qualitative study |
| 2017 | ***Beecham et al.*** Keeping all options open: Parents' approaches to advance care planning. Health Expect 2017;20:75-684. | Qualitative study |
| 2017 | ***Cicero-Oneto et al.*** Decision-making on therapeutic futility in Mexican adolescents with cancer: a qualitative study. BMC Med Ethics 2017;18:74. | Qualitative study |
| 2017 | ***Edwards et al.*** Decisions around Long-term Ventilation for Children. Perspectives of Directors of Pediatric Home Ventilation Programs. Ann Am Thorac Soc 2017;14:1539-47. | Qualitative study |
| 2017 | ***Henderson et al.*** Preparing Pediatric Healthcare Professionals for End-of-Life Care Discussions: An Exploratory Study. J Palliat Med 2017;20:662-6. | Qualitative study |
| 2017 | ***Kelly et al.*** Identifying a conceptual shift in child and adolescent-reported treatment decision making: "Having a say, as I need at this time". Pediatr Blood Cancer 2017;64***.*** | Qualitative study |
| 2017 | ***Lotz et al.*** "Hope for the best, prepare for the worst": A qualitative interview study on parents' needs and fears in paediatric advance care planning. Palliat Med 2017;31:764-71. | Qualitative study |
| 2017 | ***Odeniyi et al.*** Communication Challenges of Oncologists and Intensivists Caring for Pediatric Oncology Patients: A Qualitative Study. J Pain Symptom Manage 2017;54:909-15. | Qualitative study |
| 2016 | ***Zaal-Schuller et al.*** How parents and physicians experience end-of-life decision-making for children with profound intellectual and multiple disabilities. Res Dev Disabil 2016;59:283-93. | Qualitative study |

**Identification of additional literature**

Guidelines

| **Year** | **Bibliography** | **Study design** |
| --- | --- | --- |
| 2022 | ***Nederlandse vereniging voor kindergeneeskunde.*** Erytrocytentransfusies bij kinderen & neonaten met kanker. 2022. | Guideline paediatric palliative care |
| 2022 | ***Nederlandse Vereniging voor Kindergeneeskunde.*** Trombocytentransfusies bij kinderen met kanker. 2022. | Guideline paediatric palliative care |
| 2019 | ***Anderson et al.*** Artificial nutrition and hydration for children and young people towards end of life: consensus guidelines across four specialist paediatric palliative care centres. BMJ Supportive &amp; Palliative Care. 2019. | Guideline paediatric palliative care |
| 2016 | ***National Institute for Health and Care Excellence.*** End of life care for infants, children, and young people with life-limiting conditions: planning and management. London: NICE; 2016 (update 2019). | Guideline paediatric palliative care |
| 2016 | ***Flank J et al.*** Guideline for the Treatment of Breakthrough and the Prevention of Refractory Chemotherapy-Induced Nausea and Vomiting in Children With Cancer. Pediatr Blood Cancer. 2016;63(7):1144-51. | Guideline paediatric palliative care |
| 2014 | ***Dupuis LL et al.*** Guideline for the prevention and treatment of anticipatory nausea and vomiting due to chemotherapy in pediatric cancer patients. Pediatr Blood Cancer. 2014;61(8):1506-12. | Guideline paediatric palliative care |
| 2021 | ***Nederlandse Vereniging voor Psychiatrie.*** Multidisciplinaire richtlijn pediatrisch delier (PD) en emergence delier (ED). 2021***.*** | Guideline general paediatrics |
| 2020 | ***Nederlandse Vereniging voor Neurologie.*** Epilepsie. 2020. Available from:  <https://epilepsie.neurologie.nl/cmssite7/index.php?pageid=681> | Guideline general paediatrics |
| 2019 | ***National Institute for Health Care and Excellence.*** Depression in Children and Young People: identification and mangement. [Internet]. London: NICE; 2019. Available from: [www.nice.org.uk/guidance/ng134](https://prinsesmaximacentrum-my.sharepoint.com/personal/k_c_vanteunenbroek_prinsesmaximacentrum_nl/Documents/PAZO%20richtlijn/Methods%20paper/Appendices/www.nice.org.uk/guidance/ng134). | Guideline general paediatrics |
| 2019 | ***National Institute for Health and Care Excellence.*** Epilepsies: diagnosis and management. London 2019. Available from: [www.nice.org.uk/guidance/cg137](http://www.nice.org.uk/guidance/cg137) | Guideline general paediatrics |
| 2019 | ***Federatie Medisch Specialisten.*** Bloedtransfusiebeleid. 2019. | Guideline general paediatrics |
| 2019 | ***Nederlandse Vereniging voor Kindergeneeskunde.*** Somatisch onvoldoende verklaarde lichamelijke klachten (SOLK) bij kinderen 2019. Available from:  <https://assets.nvk.nl/p/491522/files/SOLK.pdf>. | Guideline general paediatrics |
| 2016 | ***Bolier L et al****.* JGZ-Richtlijn Angst [Internet]: Nederlands Centrum Jeugdgezondheid; 2016. Available from: <https://www.ncj.nl/richtlijnen/allerichtlijnen/richtlijn/angst>. | Guideline general paediatrics |
| 2016 | ***Oud M et al.*** JGZ-richtlijn Depressie. Nederlands Centrum Jeugdgezondheid; 2016. Available from: <https://www.ncj.nl/richtlijnen/alle-richtlijnen/richtlijn/depressie>. | Guideline general paediatrics |
| 2012 | ***National Institute for Health and Care Excellence. Spasticity*** in under 19s: Management. [Internet]. London: NICE; 2012. Available from: [www.nice.org.uk/guidance/cg145](https://prinsesmaximacentrum-my.sharepoint.com/personal/k_c_vanteunenbroek_prinsesmaximacentrum_nl/Documents/PAZO%20richtlijn/Methods%20paper/Appendices/www.nice.org.uk/guidance/cg145). | Guideline general paediatrics |
| 2012 | ***Nederlands Centrum Jeugdgezondheid.*** Huidafwijkingen, taakomschrijving en richtlijn voor de preventie, signalering, diagnostiek, begeleiding, behandeling en verwijzing. 2012. Available from: <https://assets.ncj.nl/docs/d3452a1b-34b2-4154-8106-9977ef5426f3.pdf>. | Guideline general paediatrics |
| 2010 | ***Joanna Briggs Institute.*** Effectiveness of non-pharmacological pain management in relieving chronic pain for children and adolescents. Best Practice: evidence-based information sheets for health professionals. 2010;14(17):1-4. | Guideline general paediatrics |
| 2022 | ***Integraal Kankercentrum Nederland.*** Richtlijn Jeuk in de palliatieve fase. 2022. Available from: <https://palliaweb.nl/richtlijnen-palliatieve-zorg/richtlijn/jeuk>. | Guideline adult palliative care |
| 2022 | ***Integraal Kankercentrum Nederland.*** Richtlijn palliatieve sedatie. 2022. Available from: <https://palliaweb.nl/richtlijnen-palliatieve-zorg/richtlijn/palliatieve-sedatie>. | Guideline adult palliative care |
| 2021 | ***Verpleegkundigen & Verzorgenden Nederland.*** Richtlijn Decubitus. 2021. | Guideline adult palliative care |
| 2019 | ***Integraal Kankercentrum Nederland.*** Vermoeidheid bij kanker in de palliatieve fase (3.0). 2019. Available from: [www.pallialine.nl/vermoeidheid](http://www.pallialine.nl/vermoeidheid). | Guideline adult palliative care |
| 2018 | ***Verpleegkundigen & Verzorgenden Nederland.*** Smetten (Intertrigo) preventie en behandeling. 2018. Available from: <https://www.venvn.nl/media/n0fppki5/richtlijnsmetten-september-2018.pdf>. | Guideline adult palliative care |
| 2016 | ***Nederlandse Vereniging van Revalidatieartsen.*** Cerebrale en/of spinale spasticiteit: VRA; 2016. | Guideline adult palliative care |
| 2015 | ***Integraal Kankercentrum Nederland.*** Dyspneu in de palliatieve fase (3.0). 2015. Available from: [www.pallialine.nl/dyspneu-in-de-palliatieve-fase](http://www.pallialine.nl/dyspneu-in-de-palliatieve-fase). | Guideline adult palliative care |
| 2015 | ***National Institute for Health and Care Excellence.*** Care of dying adults in the last days of life. London: NICE; 2015. Available from [www.nice.org.uk/guidance/ng31](http://www.nice.org.uk/guidance/ng31). | Guideline adult palliative care |
| 2014 | ***Integraal Kankercentrum Nederland.*** Misselijkheid en braken (4.0). 2014. Available from: www.pallialine.nl/misselijkheid-en-braken. | Guideline adult palliative care |
| 2011 | ***Integraal Kankercentrum Nederland.*** Decubitus (2.0). 2011. Available from: [www.pallialine.nl/decubitus](https://prinsesmaximacentrum-my.sharepoint.com/personal/k_c_vanteunenbroek_prinsesmaximacentrum_nl/Documents/PAZO%20richtlijn/Methods%20paper/Appendices/www.pallialine.nl/decubitus). | Guideline adult palliative care |
| 2010 | ***Integraal Kankercentrum Nederland.*** Oncologische Ulcera 2010. Available from:  <https://www.pallialine.nl/oncologische-ulcera> | Guideline adult palliative care |
| 2010 | ***Integraal Kankercentrum Nederland.*** Hoesten (2.0). 2010. Available from: [www.pallialine.nl/hoesten](http://www.pallialine.nl/hoesten). | Guideline adult palliative care |
| 2010 | ***Integraal Kankercentrum Nederland.*** Zorg in de stervensfase (1.0). 2010. Available from: <https://www.pallialine.nl/stervensfase>. | Guideline adult palliative care |

Textbooks

| **Year** | **Bibliography** | **Study design** |
| --- | --- | --- |
| 2012 | ***Goldman A et al.*** Oxford Textbook of Palliative Care for Children. 2nd ed. Oxford: Oxford University Press; 2012. | Textbook paediatric palliative care |
| 2011 | ***Wolfe J et al.*** Textbook of Interdisciplinary Pediatric Palliative Care: Saunders; 2011. | Textbook paediatric palliative care |

Systematic reviews

| **Year** | **Bibliography** | **Study design** |
| --- | --- | --- |
| 2020 | ***Kochen E et al.*** When a child dies: a systematic review of well-defined parent-focused bereavement interventions and their alignment with grief- and loss theories. BMC Palliative Care (2020) 19:28 | Systematic review |
| 2019 | ***Sieg SE et al.*** The Best Interests of Infants and Families During Palliative Care at the End of Life: A Review of the Literature. Adv Neonatal Care 2019 19(2):E9-e14 | Systematic review |
| 2019 | ***Dias N et al.*** A Systematic Literature Review of the Current State of Knowledge Related to Interventions for Bereaved Parents. Am J Hosp Palliat Care 2019 36 (12): 1124-1133 | Systematic review |
| 2019 | ***Thornton R et al.* S**coping Review of Memory Making in Bereavement Care for Parents After the Death of a Newborn. J Obstet Gynecol Neonatal Nurs | Systematic review |
| 2018 | ***Chong PH et al.*** Perceptions of a Good Death in Children with Life-Shortening Conditions: An Integrative Review. J Palliat Med 2018 22 (6): 714-723 | Systematic review |
| 2015 | ***Lichtenhal WG et al.*** Bereavement follow-up after the death of a child as a standard of care in pediatric oncology. Pediatr Blood Cancer 2015; 62;S834-S869. | Systematic review |
| 2014 | ***Donovan LA et al.*** Hospital-based bereavement services following the death of a child: A mixed study review. Palliative Medicine 2015, Vol. 29(3) 193– 210 | Systematic review |
| 2013 | ***Stevenson M et al.*** Pediatric palliative care in Canada and the United States: a qualitative metasummary of the needs of patients and families. J Palliat Med 2013 16(5):566-77 | Systematic review |
| 2012 | ***Aschenbrenner AP et al****.* Integrative review: parent perspectives on care of their child at the end of life. J Pediatr Nurs 2012 27(5):514-22 | Systematic review |
| 2011 | **L*ongdon JV et al****.* Parental perceptions of end-of-life care on paediatric intensive care units: a literature review. Nurs Crit Care 2011 16(3):131-9 | Systematic review |
